# Vascular ageing: moving from bench towards bedside

**DOI:** 10.1093/eurjpc/zwad028

**Published:** 2023-02-04

**Authors:** Rachel E. Climie, Jordi Alastruey, Christopher C. Mayer, Achim Schwarz, Agne Laucyte-Cibulskiene, Julija Voicehovska, Elisabetta Bianchini, Rosa-Maria Bruno, Peter H. Charlton, Andrea Grillo, Andrea Guala, Magid Hallab, Bernhard Hametner, Piotr Jankowski, Karsten Königstein, Anna Lebedeva, Ioana Mozos, Giacomo Pucci, Houry Puzantian, Dimitrios Terentes-Printzios, Gunay Yetik-Anacak, Chloe Park, Peter M. Nilsson, Thomas Weber

**Affiliations:** 1 Menzies Institute for Medical Research, University of Tasmania, 17 Liverpool St, 7000 Hobart, Australia; 2 Sports Cardiology, Baker Heart and Diabetes Institute, 99 Commercial Rd, Melbourne 3000, Australia; 3 Integrative Epidemiology of Cardiovascular Disease, Université de Paris, INSERM, U970, Paris Cardiovascular Research Center (PARCC), 56 rue Leblanc, 75015 Paris, France; 4 Department of Biomedical Engineering, School of Biomedical Engineering and Imaging Sciences, King’s College London, 249 Westminster Bridge Rd, London SE1 7EH, UK; 5 Medical Signal Analysis, Center for Health & Bioresources, AIT Austrian Institute of Technology, Giefinggasse 4, 1210 Vienna, Austria; 6 ALF Distribution GmbH, Stephanstrasse 19, 52064 Aachen, Germany; 7 Department of Clinical Sciences, Lund University, Skane University Hospital, Sölvegatan 19 - BMC F12, 221 84 Lund, Malmö, Sweden; 8 Faculty of Medicine, Vilnius University, M. K. C iurlionio g. 21, 03101 Vilnius, Lithuania; 9 Department of Internal Diseases, Riga Stradins University, Dzirciema str. 16, Riga, L-1007, Latvia; 10 Nephrology and Renal Replacement Therapy Clinics, Riga East University Hospital, Hipokrata str. 2, Riga, LV-1079, Latvia; 11 Institute of Clinical Physiology, Italian National Research Council (CNR), Via Moruzzi, 1, 56124 Pisa (PI), Italy; 12 Department of Public Health and Primary Care, University of Cambridge, Strangeways Research Laboratory, 2 Worts Causeway, Cambridge CB1 8RN, UK; 13 Medicina Clinica, Department of Medicine, Surgery and Health Sciences, University of Trieste, Strada di Fiume 447, 34149 Trieste, Italy; 14 Vall d’Hebron Institut de Recerca (VHIR), Paseo de la Vall d’Hebron, 129, 08035 Barcelona, Spain; 15 Clinique Bizet, 23 Georges Bizet, 75116 Paris, France; 16 Department of Internal Medicine and Geriatric Cardiology, Centre of Postgraduate Medical Education, 231 Czerniakowska St., 00-416 Warsaw, Poland; 17 Department of Sport, Exercise and Health (DSBG) University of Basel, Grosse Allee 6, 4052 Basel, Switzerland; 18 Department of Internal Medicine and Cardiology, Dresden Heart Centre, Dresden University of Technology, Fetscher str. 76, 01307 Dresden, Germany; 19 Department of Functional Sciences-Pathophysiology, Center for Translational Research and Systems Medicine, ‘Victor Babes’ University of Medicine and Pharmacy, T. Vladimirescu Street 14, 300173 Timisoara, Romania; 20 Unit of Internal Medicine, Terni University Hospital - Department of Medicine and Surgery, University of Perugia, Terni, Italy; 21 Hariri School of Nursing, American University of Beirut, P.O. Box 11-0236, Riad El Solh 1107 2020, Beirut, Lebanon; 22 First Department of Cardiology, Hippokration Hospital, Medical School, National and Kapodistrian University of Athens, 114 Vasilissis Sofias Avenue, 11527 Athens, Greece; 23 Department of Pharmacology, Faculty of Pharmacy, Acibadem Mehmet Ali Aydinlar University, Kayisdagi Cad. No:32 Atasehir, 34752 Istanbul, Turkey; 24 MRC Unit for Lifelong Health and Ageing at UCL, 1-19 Torrington Place, London WC1E 7HB, UK; and; 25 Cardiology Department, Klinikum Wels-Grieskirchen, Grieskirchnerstrasse 42, 4600 Wels, Austria

**Keywords:** Ageing, Vascular damage, Cardiovascular disease prevention

## Abstract

Prevention of cardiovascular disease (CVD) remains one of the largest public health challenges of our time. Identifying individuals at increased cardiovascular risk at an asymptomatic, sub-clinical stage is of paramount importance for minimizing disease progression as well as the substantial health and economic burden associated with overt CVD. Vascular ageing (VA) involves the deterioration in vascular structure and function over time and ultimately leads to damage in the heart, brain, kidney, and other organs. Vascular ageing encompasses the cumulative effect of all cardiovascular risk factors on the arterial wall over the life course and thus may help identify those at elevated cardiovascular risk, early in disease development. Although the concept of VA is gaining interest clinically, it is seldom measured in routine clinical practice due to lack of consensus on how to characterize VA as physiological vs. pathological and various practical issues. In this state-of-the-art review and as a network of scientists, clinicians, engineers, and industry partners with expertise in VA, we address six questions related to VA in an attempt to increase knowledge among the broader medical community and move the routine measurement of VA a little closer from bench towards bedside.

## Introduction

Cardiovascular disease (CVD) is the leading cause of death worldwide, with one in three deaths being attributable to CVD.^1^ By 2030, it is expected that CVD will cost US$1044 billion globally.^2^ Thus, prevention of CVD is a public health priority and identifying individuals at increased cardiovascular risk at an asymptomatic, sub-clinical stage is of paramount importance for minimizing disease progression as well as health and economic burden.

Vascular ageing (VA) is a process that can capture the early (generally asymptomatic) features of vascular degeneration.^3^ Given that a measure of VA encompasses the cumulative effect of all cardiovascular risk factors on the arterial wall over the life course, compared to more traditional risk factors which may fluctuate in time, a measure of VA may help identify those at elevated cardiovascular risk.

Although the concept of VA is gaining interest, it is seldom measured in routine clinical practice. This is potentially a missed opportunity to identify at-risk individuals at an early stage of disease progression. To address this, VascAgeNet^4^ is actively working to refine and harmonize measures of VA in an interdisciplinary, international, and inter-sectorial approach. In this review and as a network of scientists, clinicians, engineers, and industry partners with expertise in VA,^5^ we address six questions related to VA in an attempt to increase knowledge among the broader community and move a little closer from bench towards bedside.

## What is vascular ageing?

**Figure 1:**
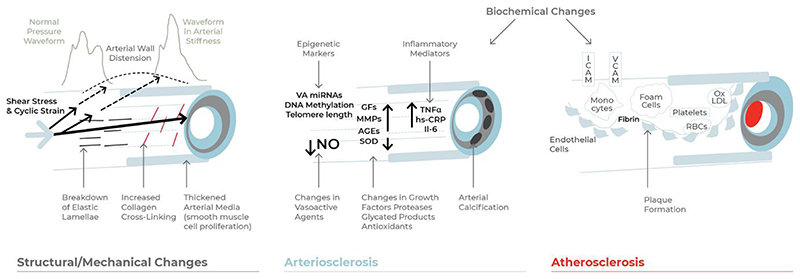
Mechanisms of vascular ageing comprising of arteriosclerotic and atherosclerotic processes. The figure depicts structural and mechanical changes, as well as major biochemical derangements contributing to vascular ageing processes. VA miRNAs, micro-ribonucleic acids of vascular ageing; NO, nitric oxide; GF, growth factors; MMP, matrix metalloproteinase; AGEs, advanced glycation end-products; SOD, superoxide dismutase; TNF-α, tumour necrosis factor-alpha; hs-CRP, high-sensitivity C-reactive protein; Il-6, interleukin-6; ICAM, intercellular adhesion molecule; RBCs, red blood cells; Ox LDL, oxidized low-density lipoprotein.

While there is no universally agreed definition for VA, it involves the deterioration in arterial structure and function over time, which ultimately leads to damage of the heart, brain, kidney, and other organs. VA includes a large spectrum of alterations affecting the functional and structural components of the arterial wall irrespective of size, traditionally included in the definitions of *atherosclerosis* and *arteriosclerosis^6^ (Figure 1).* Arteriosclerosis involves primarily the tunica media and is associated with replacement of elastin fibres with stiffer collagen, destruction of muscle fibres, and formation of calcium deposits in the media. Vessel wall changes lead to an increase in arterial stiffness with an associated increase in premature wave reflections and a decline in the buffering capacity to pulsatile arterial blood flow, which has consequences for cardiovascular health. These include: (i) elevated pulse pressure (PP) and development of isolated systolic hypertension^7^; (ii) increased left ventricular late systolic afterload, leading to ventricular re-modelling and hypertrophy, diastolic dysfunction, impaired exercise capacity, and, in the long-term, the risk of new-onset heart failure^8^; (iii) lower diastolic blood pressure (BP), in turn reducing coronary perfusion pressure^9^ and increasing the risk of coronary events^10,11^; and (iv) increased transmission of elevated pulsatile pressure/flow to the micro-vasculature of target organs.^12^ This may be particularly pertinent to organs such as the brain and kidneys which have a high demand for blood flow and, therefore, have low resistance.^13,14^ The clinical consequences include small artery re-modelling and damage in the brain^15^ (leading to leucoaraiosis and cognitive impairment/dementia) and progression of chronic kidney disease.^16^

In atherosclerosis, inflammatory and immune cells, smooth muscle cells, lipids, and connective tissue progressively accumulate in the intima of large and medium size arteries. Atherosclerotic plaques typically develop over several decades, leading to progressive narrowing of the arterial lumen. In a final step, often in combination with local thrombotic phenomena (‘atherothrombosis’), obstruction of the lumen and clinical events occur. Although the initial atherosclerotic plaques as well as plaques with large necrotic core and thin fibrous cap (so-called ‘unstable plaques’) are not stiff and their presence may be associated with reduced local arterial stiffness, the more mature plaques, especially those calcified, increase arterial stiffness significantly.^17^ Likely more important, both atherosclerosis and arteriosclerosis are systemic diseases and linked to each other, both anatomically^18^ and functionally.^19^ Furthermore, the progression in atherosclerosis is often paralleled by the progression in arterial stiffness.^20^ In fact, a bi-directional relationship between arteriosclerosis and atherosclerosis may exist whereby increased arterial stiffness contributes to progression of atherosclerosis, which in turn increases stiffness of vessel walls.^21,22^ The hypothesis that increased stiffness can lead to progression of atherosclerosis is based on the concept that stiffening-induced haemodynamic changes sensed by endothelial cells as well as strain changes sensed by smooth muscle cells and other cells in the arterial wall, including macrophages, result in pro-atherosclerotic downstream signalling events.^22,23^ It should, however, be mentioned that some studies^22,23^ suggest a mono-directional link between arterial stiffness and atherosclerosis: increased stiffness would simply be a consequence of the pathological changes that occur in the arterial wall during the progression of atherosclerosis.^23^

VA involves arterial degeneration and hardening that impairs vascular function and leads to target organ damage in the heart, brain, and kidneys.

## How can vascular ageing be estimated and what does vascular ageing add to the established biomarkers in the clinic?

**Figure 2:**
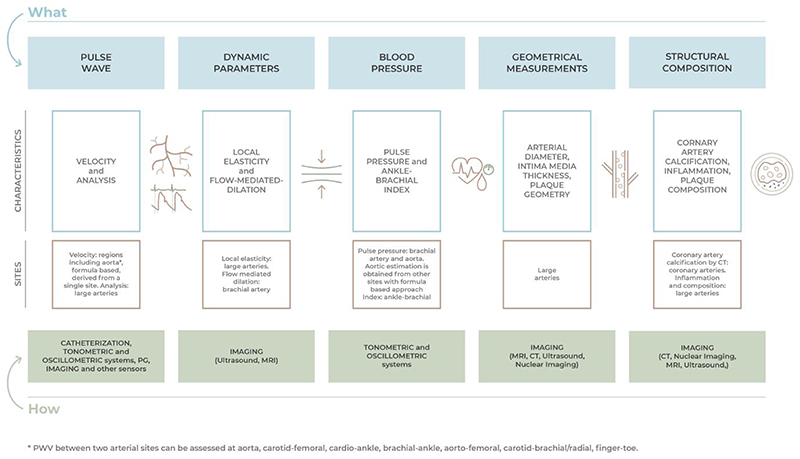
How to measure vascular age. The vascular ageing process can impact different arterial characteristics: pulse wave velocity and features, arterial dynamic or geometrical parameters, pulse pressure, and vessel structural composition. Alterations of these properties can be assessed by processing images or signals obtained using various technologies. CAC, coronary artery calcification; CT, computed tomography; MRI, magnetic resonance imaging; PG, plethysmography.

Many potential invasive and non-invasive biomarkers have been proposed within the last decades as indicators of VA. Circulating biomarkers measurable in blood and urine are an attractive target of current research trying to connect molecular processes underlying VA with clinical outcomes.^24^ However, none of these parameters currently meet the criteria for clinical application.^25^ Therefore, we focus on non-invasive parameters that (i) have already been shown to predict clinical CVD and (ii) are subject to age-related changes. These parameters are summarized in Figure 2 and detailed in Table 1. Further details can be found in the Supplementary material online. Selected clinical and prognostic information and their added value are presented if available.

Despite the availability of many parameters to estimate VA, consensus on how to categorize VA as physiological vs. pathological is still not reached and eagerly required. Following the concept that arterial damage reflects the net result of all harmful influences on the arterial wall over the life course, a dedicated measure of atherosclerosis [e.g. coronary artery calcification (CAC)] or arteriosclerosis (most often measured by pulse wave velocity) alone may quantify an individual’s VA.^58^ One option to quantify VA is in analogy with (brachial) BP: although age-related changes clearly can be shown,^59^ the threshold set between normal and elevated is fixed at a certain value and, thus, is not age-dependent.^60^ Likewise, normal or healthy VA can be defined as the absence of CAC (i.e. CAC score of zero) or, with regard to carotid-to-femoral PWV (cfPWV), as less than the 10th percentile of a young (<30 years) population with normal BP.^61^ As another option and considering the age-dependent changes of the measurements when using cfPWV, the age quintile-specific 10th percentile (for healthy VA) and 90th percentile [for early VA (EVA)] of the population^62^ have been proposed. Further, based on cardiovascular risk factors and CAC score^63^ or cfPWV,^64^ VA can be calculated with regression models, and the difference from chronological age assessed. This method offers new insights by identifying individuals in whom VA is delayed despite the presence of classical cardiovascular risk factors [so-called super-normal vascular ageing (SUPERNOVA)]. Outcome-based approaches have been reported as well^65^; these assign a VA that has the same expected coronary heart disease risk as the observed level of a measure of VA, for instance CAC. However, the superiority of age-specific thresholds, as compared to fixed cut-off values, cannot be automatically assumed: absolute CAC in standard groups (CAC score 0, 1–100, 101–400, >400) performed better for cardiovascular risk prediction than age-specific percentiles.^66^ Finally, the combination of a measure of atherosclerosis [intima–media thickness (IMT)] and a measure of arteriosclerosis (cfPWV) into a ‘vascular ageing index’ had good prognostic performance and improved cardiovascular risk prediction, as compared to IMT or cfPWV alone.^67^ With these measurements, biological mechanisms of CVD can be better understood, and the prognosis of CVD can be improved based on early prevention.

VA can be estimated by isolated or integrated measures of morphological (structural) or functional (mechanical) properties and may improve cardiovascular risk prediction.

## How do vascular ageing measures relate to chronological ageing?

**Figure 3:**
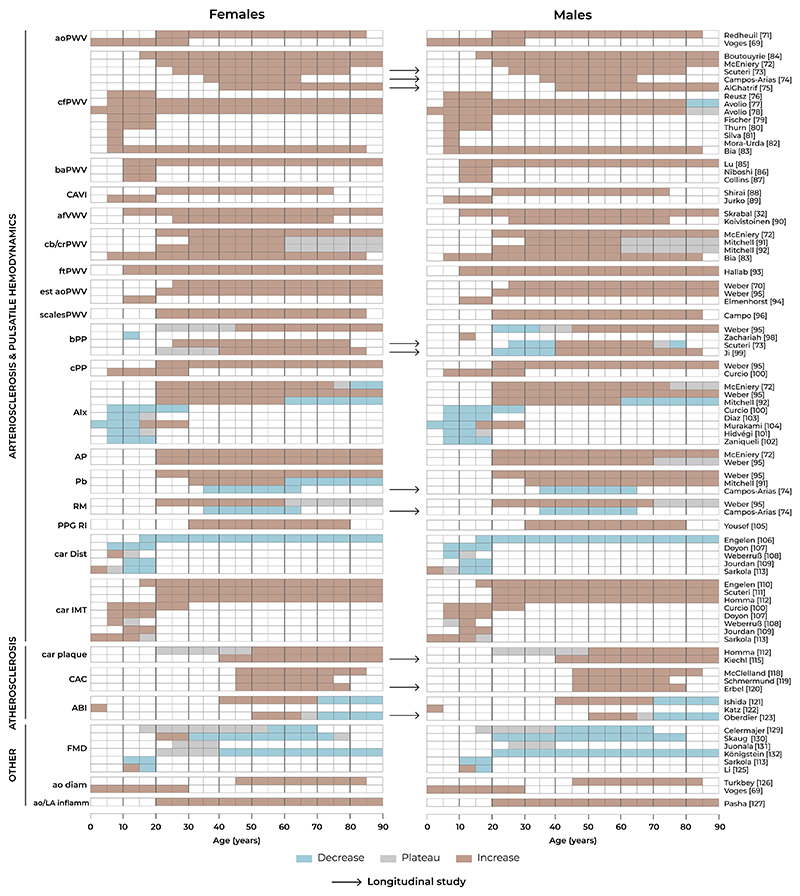
Relationship between vascular ageing measures with chronological ageing. aoPWV, aortic pulse wave velocity; cfPWV, carotid-femoral pulse wave velocity; baPWV, brachial-ankle pulse wave velocity; CAVI, cardio-ankle vascular index; afVWV, aorto-femoral volume wave velocity; cb/crPWV, carotid-brachial/radial PWV; ftPWV, finger-to-toe pulse wave velocity; est aoPWV, estimated aortic pulse wave velocity; scalesPWV, pulse wave velocity derived with bathroom scales; bPP, brachial pulse pressure; cPP, central pulse pressure; AIx, augmentation index; AP, augmentation pressure; Pb, backward wave amplitude; RM, reflection magnitude; PPG RI, photoplethysmogram-based reflection index; car Dist, carotid artery distensibility; car IMT, carotid intima-media thickness; car plaque, carotid plaque; CAC, coronary artery calcification; ABI, ankle-brachial index; FMD, flow-mediated dilation; ao diam, aortic diameter; ao/LA inflamm, large artery inflammation (positron emission tomography).

All VA parameters show distinct changes from early life to advanced age. In Figure 3, we provide details of how VA parameters relate to chronological ageing. Further details on other VA parameters (carotid artery distensibility, ankle–brachial index, aortic diameter, aortic/large artery inflammation) can be found in the Supplementary material online as well as a summary of the amalgamated literature in the Supplementary material online, Table S1.

Although it is now widely accepted that VA starts in newborns or—in the presence of unfavourable *in utero* conditions—even earlier, vascular measurements during childhood and adolescence are influenced not only by VA, but mainly from physiological growth and maturation.^68^ It is important to realize that these processes co-exist and cause typical but different changes of the various vascular measurements in the first two decades of life. Currently, due to limited longitudinal data, it is difficult to disentangle the changes that occur in the vasculature that are due to growth compared to that which is due to ageing *per se.* However, discussion of the development of the (cardio-) vascular system is beyond the scope of this review. Briefly, to accommodate the perfusion needs of the developing body, vascular wall and lumen dimensions change (expand) as the child grows.^69^ These changes also affect arterial compliance—the early growth phase is associated with increasing buffering capacity of the large arteries. However, this is not uniformly reflected by measures of VA (see below).

### Pulse wave velocity

Invasive aortic PWV,^70^ magnetic resonance imaging (MRI)-based aortic arch PWV,^69,71^ cfPWV,^72–84^ brachial–ankle PWV,^85–87^ cardio–ankle vascular index,^88,89^ aorto–femoral volume wave velocity,^32,90^ carotid–brachial/radial PWV,^72,83,91,92^ finger–toe PWV,^93^ estimated aortic PWV,^70,94,95^ and PWV from bathroom scales^96^ all increase with age in a non-linear way, with more marked changes after the age of 50 years, but beginning to increase already in childhood.^32,76–83,86,87,89,93,94^ These changes are apparent in cross-sectional studies, but are even better delineated in longitudinal studies, where it is clear that the rate of change of PWV accelerates. In one study with a follow-up duration of almost 10 years, the average rate of change in PWV increased by ≈60% from entry age of 30 years to entry age of 70 years.^73^ This was confirmed recently in a study in middle-aged healthy individuals with a follow-up duration of roughly 10 years^74^: cfPWV increased with time, and the rate of change accelerated with age, particularly in women. In contrast, changes of carotid–brachial PWV with age are small,^91,92^ particularly in men and after 60 years in both sexes. Cardio–ankle vascular index increases with age in both sexes equally.^97^ For estimation of VA, age-related population-based cut-off values, representing 10th and 90th percentiles, are available.^72,84^

### Pulse pressure

In children, brachial PP increases slowly in boys from 8 to 17 years, but plateaus in girls of the same age.^98^ In adults, brachial PP increased consistently from middle age^95,99^ in cross-sectional analysis. In contrast, central PP increased across all age groups,^95^ starting in childhood.^100^ In longitudinal studies of brachial PP,^73,99^ the increase in brachial PP was higher in women than in men. In one study,^73^ the longitudinal rate at which PP changed over time plateaued in elderly men and even declined in the oldest age group, whereas in women, the rate of change in PP increased in all age groups. A brachial PP ≥60 mmHg is a measure of hypertension-mediated organ damage in older people^60^ and age-related percentiles for central PP are available.^95^

### Waveform features related to wave reflection

In different cross-sectional studies in children, augmentation index (AIx) decreases consistently up to 15 years, with minor differences between the studies^101,102^ thereafter: further decrease up to 28 years,^100^ a plateau up to 22 years in women,^103^ and a plateau up to 25 years in the entire study population.^104^ In cross-sectional studies in adults,^72,91,92,95^ both AIx and augmentation pressure were significantly and positively correlated with age, and values were higher in women than in men at each decade of life. Whereas the association between age and augmentation pressure was linear, changes in AIx were non-linear and more prominent in those under 50 years of age.^72^ In other studies,^95^ the plateau of AIx and augmentation pressure—or even a decline in old age^91,92^—was evident from the age of 65 years onward, which was also true for reflection magnitude. Backward wave amplitude displayed a continuous rising with no clear flattening, particularly in women.^91^ In contrast, in a longitudinal study,^74^ the reflection coefficient, reflection magnitude, and backward wave amplitude decreased during a follow-up period of 10 years in healthy middle-aged men and women. The reflection index, derived from photoplethysmography at the index finger, increased with increasing age^105^ in cross-sectional analysis. Age-related population-based reference values/percentiles for several waveform parameters are available.^95^

### Carotid intima–media thickness

In cross-sectional studies, carotid IMT increased linearly with age,^110–112^ beginning in children <1 year^113^ and almost doubles from 15 to 85 years of age. Despite this fact, a carotid IMT of >0.9 mm is considered abnormal.^25,60^

### Carotid plaque

Precursor lesions of carotid atherosclerosis (intima–media thickening) may occur as early as adolescence, but the occurrence of carotid plaques in children is limited to the presence of extreme risk factors, such as familiar hypercholesterolaemia.^114^ In the general population, the frequency of definite atherosclerotic lesions remains low until age 40 in men and onset of menopause in women (prevalence <1.0% each)^112,115^ in cross-sectional analysis. The sex difference disappears within 5 years after menopause. After the age of 40 years/the onset of menopause, the cumulative risk of having plaque increased sharply and non-linearly,^112,115^ until a plateau with a prevalence of 90% was reached at older age. Moreover, in a longitudinal study, the incidence of carotid plaque in regions free from atherosclerosis at baseline increased non-linearly with age.^115^ The simple presence of carotid plaque is a marker of VA, and even asymptomatic carotid plaque with stenosis ≥50% is considered as established cardiovascular disease.^60,116^

### Coronary artery calcification

In children, CAC scores >0 occur only in the presence of exceptional risk factors, such as end-stage renal disease.^117^ In cross-sectional studies in adults, the probability of having CAC scores >0 (detectable CAC) increased from very low levels at age 40 to high levels at age 80 (+80%) in a more or less linear fashion in women.^118^ In men, the probability of having CAC scores >0 at age 40 was higher (20–30%), and increased in a non-linear fashion with age, reaching a plateau at very high levels (+90%) at age 80.^118^ When actual CAC scores were considered, the 90th percentile curve increased in a non-linear (exponential) fashion, more quickly in higher age.^118,119^ Again, men had greater CAC scores than women. In addition, there are significant differences in CAC scores by race/ethnicity. For instance, white men and women had the highest percentiles, as compared to Hispanic, Chinese, and Black men and women, respectively.^118^ In longitudinal studies, CAC progression was faster at higher age,^120^ mainly predicted by baseline CAC score, with cardiovascular risk factors having only limited influence.^120^ In the Heinz Nixdorf Recall study, with computed tomography (CT) scans spaced 5 years apart, the incidence of newly detected CAC in men and women with CAC score of zero at baseline steadily increased with age, from 23% in men 45 to 49 years of age to 67% in the 70 to 74 years of age category. In women, new onset of CAC was seen in 15% (age 45–49 years) and 43% (age 70–74 years), respectively. Newly detected CAC was associated with systolic BP, LDL–chol-esterol, and smoking.^124^ CAC-based VA has been defined either as the presence or absence of any CAC, or based on population-based age-related percentiles.^118,119^

### Endothelial function

In children, flow-mediated dilation (FMD) showed a decrease from 10 to 18 years in one study^113^ and a minor increase from 8 to 13 years in females and to 14 years in males, followed by a decrease in both sexes until 18 years.^125^ In adults, several cross-sectional studies demonstrated highest FMD values within the third decade of life,^128^ which remained stable until the end of the fourth decade in men and until the early fifth decade in women and declined thereafter following a curvilinear trend with highest rates of decline in the sixth decade.^129^ In a larger study, FMD was highest at the age of 20 years and decreased with increasing age up to 70 years for men and 80 years for women.^130^ In another study on 2265 individuals aged 24–39 years, ageing was not associated with changes in brachial FMD.^131^ In the most recent study in 457 healthy adults, aged 20–91 years, brachial FMD remained stable in women until 40 years of age and decreased thereafter, with highest rates of yearly decline between 50 and 60 years. In men, FMD decrease followed a linear trend with slightly higher rates of yearly decline in the young compared with the older participants.^132^ With the limitation that FMD values depend on the exact measurement methodology used, a recent meta-analysis suggested 6.5% as a cut-off for ‘optimal’ endothelial function, values between 3.1 and 6.5% to be classified as ‘impaired’ endothelial function, and values below 3.1% as ‘pathological’.^133^

In summary, an individual’s vascular age may be very different to their chronological age.

## Why do some people display early vascular ageing compared to others?

**Figure 4:**
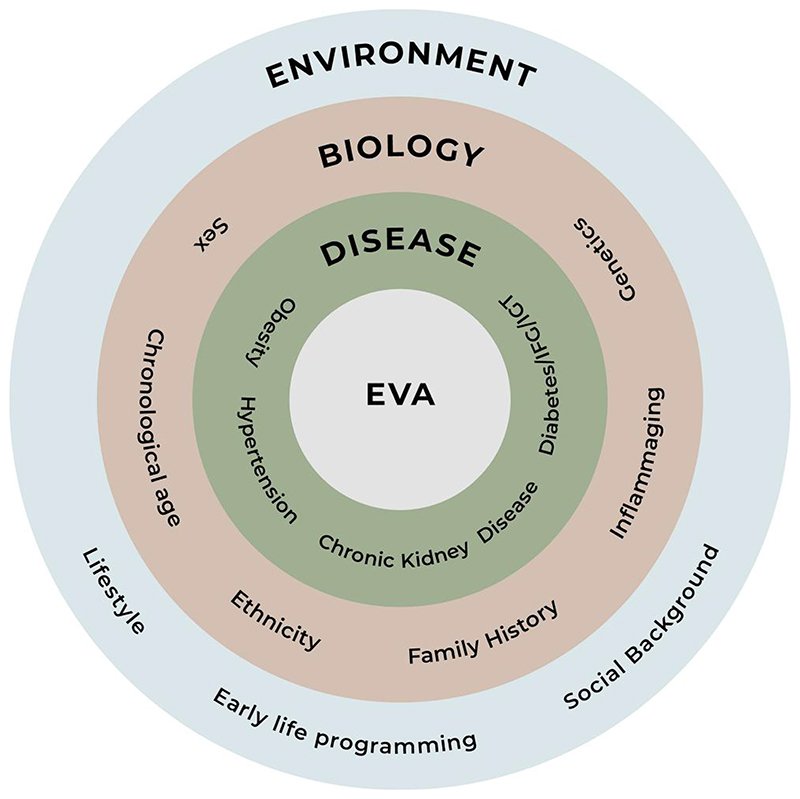
Factors contributing to why some people display early vascular ageing (EVA) compared to others. IFG, impaired fasting glucose; IGT, impaired glucose tolerance.

Conviction is increasing among scientists that biological VA is a better predictor of CVD than chronological age, leading to the introduction of the concept of early EVA.^134^ Exposure to environmental (such as CVD risk factors including smoking, obesity, hypertension, diabetes, and hypercholesterolaemia^135–139^) and genetic factors,^128^ as early as during childhood or even during foetal life,^140,141^ promotes the development and accumulation of sub-clinical vascular changes that direct an individual towards a trajectory of EVA (Figure 4). In comparison to normal arterial ageing, EVA also encompasses changes in the peripheral circulation, i.e. in the smaller arterioles, therefore enhancing the cross-talk with large elastic arteries and arteriosclerosis.^142^ The EVA phenomenon also represents a burden among offspring with a positive family history of CVD or type 2 diabetes.^143,144^ Additionally, emerging evidence concur that early life programming is also an important player in vascular re-modelling mainly because the architecture of the vascular system is programmed *in utero* and that elastin, the major structural component underlying arterial wall elasticity, is synthesized and deposited during this time.^145^ While the combination of prematurity and intrauterine growth retardation appears to be associated with the most marked impairments in vascular structure and function, the small-for-gestational-age phenotype, followed by a rapid ‘catch-up’ growth in early years, also appears harmful, the so-called mis-match condition.^146^ A joint Italian–American study provided evidence that around 40 previously identified genetic markers of hypertension did not overlap with those for PWV.^147^ However, the hunt for genes related to vascular re-modelling is ongoing and may help to explain the genetic background of different arterial wall changes, leading to arterial stiffening and other age-related features. Recently, in a UK Biobank study, a simplified measure of arterial stiffness was applied using photoplethysmography and results analysed according to genome-wide association studies findings in more than 127 000 subjects.^128^ Four loci were identified reaching genome-wide significance (*P* < 5 × 10^−8^) for association with the arterial stiffness index: *TEX41* (rs1006923; *P* = 5.3 × 10^−12^), *FOXO1* (rs7331212; *P* = 2.2 × 10^−11^), *C1orf21* (rs1930290, *P* = 1.1 × 10^−8^), and *MRVI1* (rs10840457, *P* = 3.4 × 10^−8^). Gene-based testing revealed three significant genes, and the most significant gene was *COL4A2* (*P* = 1.41 × 10^−8^) encoding type IV collagen. Other candidate genes at associated loci were also involved in smooth muscle tone regulation.^128^ Other epigenetic changes, reflecting the influence of environmental factors on the activation or silencing of genes, may be of importance for the development of arterial stiffness. These were summarized in a review by Lacolley *et al*.^148^

Ethnicity could also contribute to EVA as evident in the multi-ethnic Dallas Heart Study cohort where Afro–Americans and Hispanics, as compared to Caucasians, had stiffer proximal aorta even after adjustment for traditional cardiovascular risk factors. Across all ethnic groups, for given levels of BP and age, some people have stiffer central arteries than others, as recently reviewed.^149^ On the other hand, ethnicity should always be addressed in the context of current lifestyle and social factors. Although native Japanese subjects suffer less from atherosclerosis compared to Americans, Japanese immigrants to the USA develop a similar degree of atherosclerosis risk as the general population, thereby supporting the western lifestyle hypothesis for CVD development.^150^

Some comorbidities are likely promoters of EVA, for example uncontrolled hypertension, impaired glucose metabolism, insulin resistance, and chronic inflammation. Diabetes has been described as a model for premature VA,^139^ especially if poorly controlled. Some categories of patients with conditions characterized by chronic inflammation, such as rheumatoid arthritis and inflammatory bowel disease, are also at higher risk of vascular re-modelling as are patients with chronic kidney disease as mentioned above.

Early VA may be due to genetics, early life programming including the pre-conception period, poor diet, inactivity, and risk factors such as hypertension, hyperlipidaemia, diabetes, or obesity.

## Who benefits most from a measurement of vascular ageing?

Measurements related to VA may improve the perception of cardiovascular risk, facilitate communication with patients, and benefit adherence to therapy.^151^ Thus, such measures may be useful in primary and secondary prevention for two main reasons: first, because they are assumed to integrate the detrimental effect of traditional (BP, glycaemia, or lipids) and emergent (e.g. inflammation) cardiovascular risk factors in the unique process of VA over the lifetime, and second, because it is assumed that, contrary to single cardiovascular risk factors, measures of VA are less prone to fluctuations over time.^138^ Additionally, risk scores provide an absolute risk of events, while the concept of VA rather provides the risk of a subject compared to a peer of the same chronological age, and this can be more informative for the clinician and of greater impact for patients when discussing health status. Therefore, most individuals may benefit of VA assessment. Further, measurements related to VA may be particularly useful in special populations (i.e. in the young, elderly, or in conditions such as chronic kidney disease), in which traditional scores may fail to capture real cardiovascular risk.

International guidelines addressing the prevention of CVD and the management of arterial hypertension encourage measurements of vascular biomarkers related to VA. In the 2019 European Society of Cardiology/European Society of Atherosclerosis guidelines for the management of dyslipidaemias, significant atherosclerosis on a CT scan or carotid ultrasound automatically classifies a patient at very high risk.^152^ The 2021 European Society of Cardiology guidelines for cardiovascular prevention suggest carotid ultrasound and CAC score may be considered,^116^ while the American guidelines on the primary prevention of CVD recommend only CAC.^153^ In addition, there is evidence showing that cfPWV improves prediction of cardiovascular events and re-classification of patients at risk, especially among individuals at intermediate risk.^28^ Further studies showed that cardiovascular risk prediction can be improved by adding markers of sub-clinical organ damage (PWV, albuminuria, left ventricular hypertrophy, FMD) to SCORE^154^ or the Framingham risk score.^132^

### Vascular ageing assessment in apparently healthy people

High BP together with age is the main factor accelerating VA, in particular when assessed as arterial stiffness.^155^ Hypertension is one of the first diseases in which prognostic value for PWV was discovered^156^ and uptake of PWV into guidelines was first achieved in hypertension.^60^ Assessment of sub-clinical vascular damage by measurements related to VA (e.g. cfPWV, carotid ultrasound) may be a useful indicator for timely treatment initiation in newly diagnosed, Grade 1 hypertensive patients, by indicating the presence of hypertension-mediated organ damage, though its use is not routinely recommended in the most recent guidelines.^60^ Similarly, VA detection should mandate timely treatment of dyslipidaemia with tighter LDL–cholesterol thresholds, as for patients at very high risk.^152^ For example, VA detection could help in treatment decisions, especially in patient categories where evidence is less stringent, i.e. elderly >70 years old.^116^ Isolated systolic hypertension in the young may be a similar case, where assessment of central BP may help and is already recommended.^157^ The recent International Society of Hypertension guidelines recommend PWV assessment in the presence of isolated systolic hypertension.^158^

### Vascular ageing assessment in patients with established atherosclerotic cardiovascular disease

The predictive value of measurements associated with VA in secondary prevention has been extensively studied, especially in patients with coronary artery disease,^10,11^ although a clear benefit on outcomes still needs to be proved. Another promising application of VA biomarkers is the reduction of unnecessary invasive tests, such as coronary angiography. Recent data suggest that when combing measures of VA with artificial intelligence algorithms, coronary artery disease can be accurately and non-invasively detected in individuals with suspected coronary artery disease.^159,160^ An eight-fold risk for suffering a cardiovascular event or death has been demonstrated in patients with accelerated VA captured with a combination of biomarkers (brachial–ankle PWV and flow-mediated dilation).^161^

### Vascular ageing assessment in patients with risk modifiers

VA biomarkers may be particularly useful in conditions in which traditional risk scores are not applicable. As discussed above, VA biomarkers can be used to identify children and adolescents at risk (i.e. with positive family history or presence of a specific risk factor) and to track improvement in lifestyle during youth.^68,162^ Furthermore, measuring central rather than brachial BP might be particularly beneficial in youth, since central BP is more tightly correlated with cardiac and vascular damage,^163,164^ thus being an interesting tool in children with hypertension.^162,165^

In patients with chronic kidney disease or end-stage renal disease, increased estimated arterial stiffness is independently associated with worse outcomes^166^ and is able to re-classify cardiovascular risk.^167^ Furthermore, patients with end-stage renal disease in which PWV is not reduced by BP-lowering treatment showed a higher cardiovascular mortality than their counterparts.^168^ Central BP seems to be a promising risk predictor in chronic kidney disease patients too,^169,170^ although there are ongoing discussions related to accuracy^171^ and calibration methods.^172,173^

In genetic disorders with potentially fatal cardiovascular manifestations, as Marfan syndrome, arterial stiffness measurement may be able to evaluate the risk of aortic dilatation and dissection.^174,175^ The measurement of central PP^176^ and of aortic mechanical properties by advanced MRI^177^ may further refine their risk of vascular complications and response to treatment.

In patients with chronic inflammation, such as inflammatory bowel syndrome or systemic connective tissue diseases, characterized by dis-proportionally increased cardiovascular risk, the use of measurements related to VA to correctly stratify risk is promising, although at present no specific studies have been conducted to date.^178^

In summary, many people will benefit from the measurement of VA, but in particular, patients at intermediate risk or with special conditions may benefit more from risk re-classification in their clinical and therapeutic management.

## How can vascular ageing be modified?

While some risk factors are non-modifiable, such as chronological age, sex, ethnicity, and genetics, others can be modified. Against the above background, preventive maternal and child health care may be of great importance to safeguard health conditions in early life and thereby support prevention of EVA and CVD.

### Lifestyle modifications

A healthy lifestyle has been reported to partly mitigate genetic risk for atherosclerotic events.^179^ A sedentary lifestyle contributes to atherosclerosis and arterial wall stiffening, enabled by oxidative stress.^180^ Physical activity is an effective intervention for minimizing accelerated arterial stiffness, especially moderate aerobic exercise and high-intensity intermittent training or combined resistance as well as potentially yoga and static stretching exercise.^181–188^ Aerobic exercise training increased carotid artery compliance and decreased the β-stiffness index, correlated with the changes in plasma Klotho concentration.^185^ Pierce *et al*.^186^ demonstrated a significant reduction in augmentation index following acute aerobic exercise in healthy individuals. Low-to-moderate-intensity resistance exercise effectively improves arterial stiffness.^189^ Mechanisms involve lowering of oxidative stress and serum lipids and the increase in endothelial nitric oxide bioavailability.^181,190^ Longitudinal and interventional studies suggest that increased physical activity in early life has beneficial impacts on some markers of VA.^191–194^ Indeed, ideal cardiovascular health (as defined by the American Heart Association) has been shown to be inversely related to PWV in adolescents^195^ and in younger (30–39 years) and slightly older (42–45 years) adults.^162^

Exercise should be accompanied by a healthy diet, rich in fruits and vegetables that minimize premature progression of VA via antioxidant and anti-inflammatory effects, improved endothelial function and lipid profile.^196–203^ The beneficial effects of dietary or antioxidant supplementation on VA have been observed even from early life.^204–206^ Lycopene, the unsaturated carotenoid, found in red-coloured fruits and vegetables, especially tomatoes and watermelon, may be favourable for vascular ageing due to its anti-atherosclerosis, antioxidant, anti-inflammatory, antihypertensive, antiplatelet, antiapoptotic, protective endothelial effects, and the ability to improve the metabolic profile.^200^ Resveratrol, a naturally occurring polyphenol, found mostly in the skin of red grapes, peanuts, and several types of berries may protect arterial function, given its antioxidant effect, stimulation of autophagy, the increase of nitric oxide in endothelial cells, the decrease of sodium re-absorption and serum angiotensin II level, and reducing blood pressure.^201–203^ A healthy vascular diet should also comprise of polyunsaturated fatty acids,^207–209^ cocoa flavonoids,^210–213^ tea catechins,^214,215^ and dairy products,^216,217^ while limiting salt,^218–222^ red meats,^223–225^ caffeine,^226,227^ and alcohol consumption.^228^ Polyunsaturated fatty acids reduce synthesis of pro-inflammatory mediators, blood pressure, and LDL–cholesterol and increase availability of nitric oxide in the vascular wall, explaining its favourable effect on arterial stiffness^207–209^ and promising anti-atherogenic effects.^229^ Cocoa exerts beneficial effects on vascular function.^210–213^ Habitual tea consumption, especially green tea, may have a protective vascular effect, due to antioxidant effects of tea catechins.^214,215^ Dairy products improve endothelial function and arterial stiffness due to their mineral content and lactotripeptides^216,217^ which have a beneficial impact on lipid metabolism, inflammatory factors, and oxidative stress. The beneficial effects of vitamin supplementation on VA are also reported.^230–232^

A high sodium intake is associated with increased arterial stiffness related to endothelial dysfunction regardless of BP. Several studies revealed the association between hypokalaemia and arterial stiffness which is related to its effect on endothelial function and BP.^218–222^ A diet rich in meat is associated with increased PWV.^223–225^ The effects of chronic coffee consumption on vascular function are debated. Caffeine acutely increases arterial stiffness and negatively impacts vascular health in some studies,^226,227^ while regular consumption may be inversely associated with arterial stiffness and central and peripheral BP.^233^ Alcoholic beverages, such as red wine, beer, and vodka, may protect against oxidative stress-induced increases in arterial stiffness.^228^ Alternatively, a recent systematic review showed that while light to moderate alcohol consumption may have minimal effects on FMD, heavy alcohol consumption is associated with a decrease in FMD.^234^

### Risk factor modification

Smoking cessation, weight loss, and controlling/lowering blood glucose and BP all have beneficial effects on VA. Smokers have decreased vascular distensibility, increased arterial stiffness, and increased atherosclerosis (CAC and carotid IMT) compared to never smokers.^135,235^ The adverse changes in stiffness may be dose dependent.^236^ In ex-smokers, time since quitting is independently associated with less atherosclerosis^237^ and arteriosclerosis parameters may return to non-significant levels after a decade of smoking cessation.^238^ Obesity leads to haemodynamic alterations, chronic inflammation, and endothelial dysfunction that impair vascular structure and function. Many weight loss interventions have reported beneficial effects on the vasculature however not all significant. In a recent meta-analysis, weight loss was associated with a decline in cfPWV and brachial–ankle PWV, accompanied by simultaneous decreases in BP.^239^ In another study, carotid IMT and brachial FMD were significantly reduced 9 months after bariatric surgery.^240^ These suggest that weight loss has the potential to successfully modify VA. Restricting calories and intermittent fasting can also improve endothelial function and reduce arterial stiffness and blood pressure.^241^ Diabetes and hypertension are associated with accelerated VA and the combination of both has a particularly detrimental effect^242,243^; thus, it is important to emphasize interventions that help control plasma glucose levels and BP for improved outcomes. Mendelian randomization techniques have provided evidence of a causal association between type 2 diabetes and increased arterial stiffness, assessed as brachial-ankle PWV.^244^ In type 2 diabetes, the combination of 1 year of exercise and weight loss can promote a significant decrease in glycated hemoglobin (HbA_1c_) and cfPWV.^245^ In contrast to these findings, in a cross-over randomized trial of type 1 diabetes and type 2 diabetes, acute high-intensity aerobic exercise did not affect PWV but did induce a significant reduction in wave reflection (augmentation index) and haemodynamic responses.^246^ So far, the most important study was the randomized, controlled SPARTE study in France where a strategy aimed at reducing PWV was more successful to control arterial stiffness during follow-up than a strategy based on recommendations in guidelines for cardiovascular prevention. Increased use of antihypertensive drugs was part of the ambition to control PWV and thus more participants in the intervention group were prescribed such drugs, especially the newer classes such as blockers of the renin-angiotensin system or calcium antagonists.^247^ Among the newer glucose-lowering drugs, sodium-glucose transporter (SGLT-2) inhibitors do not decrease PWV in patients with established cardiovascular disease or cardiovascular risk factors. However, a systematic review has shown that SGLT-2 inhibitors lead to a slight, but significant decrease in PWV in patients with type 2 diabetes.^248^ This could be a direct effect but also secondary to natriuresis, weight loss, and BP reduction to name some of the mechanisms involved.

Controlling other risk factors such as lowering stress and normalizing sleep patterns may also modify VA. Gut dysbiosis related to Western diet is also associated with VA.^249,250^ Unfavourable sleep quality is associated with VA as assessed by PWV^251^ and atherosclerosis (carotid IMT).^252^ Techniques to manage stress, such as yoga,^188^ have been effective in preventing or reducing the arterial stiffness in young healthy and obese, and elderly hypertensive patients. Yoga can reduce sympathetic activity and improve endothelial function with enhancement in nitric oxide bioavailability.

### Pharmacological interventions

Despite the ample evidence that lifestyle change is beneficial for vascular health, adherence to such changes can be low; therefore, medical therapy is an attractive alternative. Various pharmacological treatments can exert beneficial effects on arterial function.^253^ These include statins, aspirin, antidiabetic,^254^–^272^ anti-inflammatory drugs, and some antihypertensive drugs such as renin–angiotensin–aldosterone system blockers.^273^–^282^ See Supplementary material online for more details. Agents that target dyslipidaemia, such as statins and proprotein convertase subtilisin/kexin type 9 (PCSK9) inhibitors, are effective in atherosclerosis stabilization and regression.^283^ Therapies to safely improve the material properties of the arterial wall can modify VA. However, many treatments target the consequences of ageing, rather than the pathophysiology.

Vascular ageing can be delayed or attenuated by adopting a healthy lifestyle including a healthy diet, regular exercise, weight loss, smoking cessation, stress management, or taking prescribed medication to manage risk factors.

## Conclusion

Current guidelines for CVD prevention predominantly recommend assessing biomarkers representing the atherosclerosis component of VA. According to the European guidelines for CVD prevention, carotid ultrasound and CAC score may be considered because of their reclassification potential in addition to traditional risk scores^116^ whereas American College of Cardiology/American Heart Association (ACC/AHA) guidelines recommend only CAC score.^153^ However, arteriosclerosis is equally relevant as a mechanism of age-related diseases. Indeed, arteriosclerosis and atherosclerosis, although intrinsically intertwined, have traditionally been investigated by separate scientific groups, which has led to the incomplete inclusion of VA into major clinical guidelines in cardiovascular medicine. In this review, we considered both processes, while highlighting the currently under-rated arterio-aspect of VA. In our view, both aspects of VA should be ultimately assessed in routine clinical practice.

## Future directions

Further studies are needed to clarify important aspects such as the best strategy to quantify VA (atherosclerosis or arteriosclerosis or ideally both) and the best interventions for EVA. It is highly likely, but still needs to be shown in randomized trials, that identification of EVA has a huge potential for improving adherence on patient’s side and inertia on the physician’s side. The numbers needed to screen for EVA and to treat EVA in order to avoid one cardiovascular event need to be established and also the cost effectiveness of such an approach. In line with this and if based on sound scientific evidence, ‘anti-(vascular) ageing’, which is quite popular among the general population, could make a difference in cardiovascular prevention.

## Supplementary Material

Supplementary Material

## Figures and Tables

**Table 1:** Method of measurement for vascular ageing biomarkers and added value to established biomarkers

Ageing biomarker	Method of measurement	Added value
Aortic pulse wave velocity	Regional measure by phase-contrast MRI; measured using time-resolved 2D or 3D MRI. Better quantification achieved in the frequency domain, pairing flow waveforms via Fourier or wavelet analysis.	Significant independent predictor of CVD events in middle-aged individuals.^26^
Carotid-femoral pulse wave velocity	Ratio of travelled distance between the carotid and femoral pulse sites and transit time between common carotid and common femoral artery; based on tonometers, piezoelectric sensors, cuffs, or Doppler ultrasound, either simultaneously or sequentially, using ECG for gating; travel distance measured at body surface.	Independent predictive value for cardiovascular events and mortality^27^; potential for re-classification of patients beyond commonly recommended risk scores^28^; current gold standard of arterial stiffness assessment.^29^
Brachial-ankle pulse wave velocity	Transit time calculated with occlusive cuffs placed at brachial artery and ankle.	Prognostic value for all-cause mortality and cardiovascular events, independent of traditional risk factors.^30^
Cardio-ankle vascular index	Cardio-ankle vascular index is a variation of brachial-ankle pulse wave velocity and measured with occlusive cuffs and phonocardiogram. It is a marker of arterial stiffness based on the stiffness parameter *β* and reflects arterial stiffness from origin of the ascending aorta to the ankle.	Prognostic value for all-cause mortality, cardiovascular mortality, and cardiovascular events. May improve risk classification.^31^
Aorto-femoral volume wave velocity	Segmental impedance plethysmography with dedicated electrodes placed at regular ECG leads plus at the right side of the neck used to derive an arterial plethysmogram for the four extremities.	Independent associations with cardiovascular risk factors and development of hypertension shown in young adults.^32^
Carotid-brachial/radial pulse wave velocity	Similar to carotid-to-femoral PWV measured as transit time and travel distance between the two measuring sites (common carotid artery in the neck and brachial/radial artery at the arm).	Aortic–brachial arterial stiffness mis-match, defined as carotid–femoral pulse wave velocity divided by carotid–radial pulse wave velocity, was an independent predictor for mortality in dialysis patients.^33^
Finger-toe pulse wave velocity	Involves photoplethysmographic probes placed at the pulpar artery of the finger and the toe.	Easy-to-use measurement device, investigator independent, a good correlation with the reference method has been published, detection algorithm has been improved and validated in adults.^34^
Estimated and formula-based pulse wave velocity	Estimation of pulse wave velocity using formulas, e.g. from the Reference Value population project based on age, systolic BP, and pulse waveform characteristics.	Independent prognostic value including significant re-classification in secondary analysis of the SPRINT trial^35^; prospective data from the MORGAM project; and in patients undergoing coronary angiography.^36^
Pulse wave velocity derived with bathroom scales	Dedicated bathroom scales measure the time delay between ventricular ejection and pulse arrival at the foot.	Estimation of the aortic pulse wave velocity is feasible with a bathroom scale, but this measure lacks formal invasive validation studies.^37^
Brachial pulse pressure	Measured using validated sphygmomanometers; brachial pulse pressure defined as systolic minus diastolic BP.	Significant predictor of heart failure and all-cause mortality in middle-aged and elderly individuals.^38^
Central pulse pressure	Central pulse pressure based on waveforms recorded at the radial, brachial, or carotid artery, mainly using tonometers or cuffs; waveforms are calibrated with brachial BP and processed with dedicated formulas (e.g. transfer functions or regression models) leading to central systolic BP and pulse pressure.	Central hypertension increased cardiovascular and cerebrovascular risk irrespective of brachial BP status in a person-level meta-analysis.^39^
Waveform features related to wave reflections	Information on wave reflection derived by pulse waveform analysis based on central waveforms, e.g. augmentation index or parameters of wave separation analyses using (measure or model-based) flow waveforms.	Indices of wave reflections are independent predictors of cardiovascular events and of heart failure, with significant risk re-classification.^40^
Photoplethysmographic assessment	Photoplethysmogram used to derive an arterial pulse wave signal and several parameters. It can be assessed at various locations such as at the finger, by pulse oximeters for example.	Association of some of the derived indices with carotid-to-femoral pulse wave velocity and the presence of peripheral arterial disease.^41^
Distensibility of large arteries	Distensibility can be estimated by a relative change in diameter, area, or volume divided by the pulse pressure generating this change; often measured as change in diameter by ultrasound or area by MRI using peripheral pressure.	Aortic distensibility predicts all-cause mortality and cardiovascular events among individuals without overt cardiovascular disease^42^; carotid distensibility is an independent predictor of cardiovascular events.^43^
Carotid intima–media thickness	Assessed as the distance between the lumen–intima interface and the media–adventitia interface at different carotid segments using computerized systems based on ultrasound data processing or echo-tracking.	Association with future CVD events in individuals at high risk; whether a change in carotid intima–media thickness relates to future event risk is controversial.^44,45^
Carotid plaque	Defined as the presence of a focal wall thickening at least 50% greater than the surrounding vessel wall or as a focal region with an intima–media thickness ≥1.5 mm protruding into the lumen; obtained by ultrasound data or computerized tomography, MRI, and nuclear imaging; contrast-enhanced ultrasound imaging to assess plaque instability.	Presence of carotid plaque and carotid plaque burden are independent predictors of cardiovascular events, and significantly improve risk re-classification.^46^
Coronary artery calcification	Measured with electron-beam computed tomography or multi-slice computed tomography, and quantified semi-automatically as Agatston score.	Coronary artery calcification is a sign of sub-clinical coronary atherosclerosis; improves accuracy of risk prediction based on the Framingham risk score.^47^
Ankle–brachial index	Ratio of ankle systolic blood BP to brachial systolic BP; assessment with cuff-based systems, or with hand-held tonometers (recommended).^a^	Measure of asymptomatic hypertension-mediated organ damage; associated with an increased risk of cardiovascular and all-cause mortality; improvement beyond Framingham risk score in general population.^48,49^
Brachial artery flow-mediated dilation	Flow-mediated dilation induces the release of nitric oxide, resulting in vasodilation that can be assessed as an index of vasomotor function; ischaemia is caused by arterial occlusion using a cuff and released after 5 min leading to reactive hyperaemia; meanwhile, the brachial artery is imaged above the antecubital fossa in the longitudinal plane, and the diameter of the artery and the vasodilatation is assessed by ultrasound.	Related to the risk of cardiovascular events; a 1% increase in flow-mediated dilation is related to a 12% reduction in cardiovascular events.^25,50–53^
Aortic diameter	Leading measure of large artery size; can be measured by ultrasound, MRI, or computed tomography.	Independent prognostic value in the general population, even at values lower than those used for clinical definition of aneurysm.^54^
Large artery inflammation (positron emission tomography)	Combined with computed tomography or magnetic resonance, positron emission tomography imaging has been applied successfully in the assessment of large arteries inflammation mainly by evaluating ^18^F-fluorodeoxyglucose (^18^F-FDG) standardized uptake values.	^18^F-FDG SUV is independently related to the occurrence of cardiovascular events^55,56^; is a promising therapeutic target.^57^

For details and further references, see Supplementary material online. BP, blood pressure; CVD, cardiovascular disease; ECG, electrocardiogram; MRI, magnetic resonance imaging.

a blood BP.
