# Supplementary material for: Vascular ageing: moving from bench towards bedside
Source: Eur J Prev Cardiol. Author manuscript; Available in PMC 2023 Aug 23. (PMC7614971; doi:10.1093/eurjpc/zwad028)
Supplement: Supplementary Material [file EMS176314-supplement-Supplementary_Material.docx]

**Vascular Ageing – Moving from Bench towards Bedside**

Climie RE^1,2,3^, Alastruey J^4^, Mayer CC ^5^, Schwarz A^6^, Laucyte-Cibulskiene A^7,8^, Voicehovska J^9,10^, Bianchini E^11^, Bruno RM^3^, Charlton P^12^, Grillo A^13^, Guala A^14^, Hallab M^15^, Hametner B^5^, Jankowski P^16^, Königsten K^17^, Lebedeva A^18^, Mozos I^19^, Pucci G^20^, Puzantian H^21^, Terentes-Printzios D^22^, Yetik-Anacak G^23^, Park C^24^, Nilsson PM^7^, Weber T^25^

On behalf of the VascAgeNet Education and Dissemination Working Group.

^1^ Menzies Institute for Medical Research, University of Tasmania, Hobart, Australia

^2^Baker Heart and Diabetes Institute, Melbourne, Australia

^3^Université de Paris, INSERM, U970, Paris Cardiovascular Research Center (PARCC), Integrative epidemiology of cardiovascular disease; Paris, France

^4^Department of Biomedical Engineering, School of Biomedical Engineering and Imaging Sciences, King’s College London, UK

^5^Medical Signal Analysis, Center for Health & Bioresources, AIT Austrian Institute of Technology; Vienna, Austria

^6^ALF Distribution GmbH, Aachen, Germany

^7^Department of Clinical Sciences, Lund University, Skane University Hospital, Malmö, Sweden

^8^Faculty of Medicine, Vilnius University, Vilnius, Lithuania

^9^Department of Internal diseases, Riga Stradins University, Latvia

^10^Renal disease and kidney replacement therapy clinics, Riga East University Hospital, Latvia

^11^Institute of Clinical Physiology, Italian National Research Council (CNR), Pisa, Italy.

^12^Department of Public Health and Primary Care, University of Cambridge, Cambridge, United Kingdom

^13^Medicina Clinica, Department of Medicine, Surgery and Health Sciences, University of Trieste, 34149 Trieste, Italy

^14^Vall d’Hebron Institut de Recerca (VHIR), Barcelona, Spain

^15^Clinique Bizet, Paris, France

^16^School of Public Health Centre of Postgraduate Medical Education, Warsaw, Poland

^17^Department of Sport, Exercise and Health (DSBG) University of Basel, Grosse Allee 6, 4052 Basel, Switzerland

^18^ Clinic of Internal Medicine and Cardiology Dresden Heart Center, Dresden University of Technology

^19^University of Medicine and Pharmacy, 300173 Timisoara, Romania.

^20^Internal Medicine Department of Medicine and Surgery, University of Perugia Terni

^21^American University of Beirut

^22^1st Department of Cardiology, Hippokration Hospital, Athens Medical School, National and Kapodistrian University of Athens, Athens, Greece.

^23^ Faculty of Pharmacy Department of Pharmacology Ege University, Bornova-Izmir, Turkey

^24^MRC Unit for Lifelong Health and Ageing at UCL, Institute of Cardiovascular Science, UCL, London, UK

^25^Cardiology Department, Klinikum Wels-Grieskirchen, Wels, Austria

**Short title:** Clinical applications of vascular ageing research

**Corresponding author:**

Dr Rachel Climie, PhD

Menzies Institute for Medical Research

17 Liverpool St, Hobart, Australia. 7000.

Tel: +61 469393867

Email: Rachel.Climie@utas.edu.au

**Word count:** 15603

**Number of tables:** 1

**Number of figures:** 4

**Sources of funding:** R.E.C is supported by the National Health and Medical Research Council of Australia (reference: 2009005) and by a National Heart Foundation Future Leader Fellowship (reference: 105636). A Gu. received funding from Spanish Ministry of Science, Innovation and Universities (IJC2018-037349-I) and from ”la Caixa” Foundation (LCF/BQ/PR22/11920008).

**Disclosures:** None

**Supplementary text**

**2. How can vascular ageing be measured and what does vascular ageing add to the established biomarkers in clinic?**

*Invasive aortic pulse wave velocity*

Aortic pulse wave velocity (PWV) from the ascending aorta to the aortic bifurcation can be measured invasively during cardiac catheterization. The measurement is highly reproducible (1), and has the advantage of being purely physiological, because transit time and travel distance are perfectly aligned (2), i.e. the pulse wave travels down in exactly the same arterial segment where transit time is assessed. Moreover, travel distance can be measured very accurately from catheter length. Invasive aortic PWV is associated with hypertensive organ damage (2), and has prognostic value in patients undergoing coronary angiography, independent not only from classical cardiovascular risk factors, but also from the extent of coronary atherosclerosis and left ventricular function (3).

*Aortic pulse wave velocity by MRI*

Aortic PWV can be measured regionally by phase-contrast MRI, a technique that allows blood speed assessment. Aortic PWV is measured using time-resolved 2D (4) or 3D MRI (5), with comparable results (4). In a recent study automatic tracking of the aorta 3-D centreline in three commonly used cardiovascular MRI sequences led to more accurate distance measurements than measurements from a 2-D oblique-sagittal plane (6). Due to relatively poor temporal resolution, the quantification of transit time may be better achieved in the frequency domain, pairing flow waveforms via Fourier or wavelet analysis (4, 7).

*Carotid-femoral pulse wave velocity*

Measurement of carotid to femoral pulse wave velocity (cfPWV) is considered the current gold standard of (regional) arterial stiffness (8). For the assessment of the transit time various sginal sources and the foot of the waveform as characteristic point can be used (9). Recently, a cuff has also been used to record femoral signals. Travelled distance needs to be measured at body surface, as direct distance between both measuring sites (multiplied by 0.8 for better agreement with true anatomical distance), or as subtracted distance (suprasternal notch-femoral minus suprasternal notch-carotid) (10). The direct distance x 0.8 method involves only one measurement and has been preferred in the Reference Value Project (11). In practice, it is important for longitudinal comparisons to always use the same method. Assessment of cfPWV takes 20-30 minutes, is operator-dependent but relatively easy and robust, but can be challenging in obesity and arrhythmia. Exposure of the groin may be a disadvantage in some cultures.

*Brachial-ankle pulse wave velocity*

With the use of plethysmographic cuffs placed at the brachial artery and the ankle, the delay between the upstroke at both sites can be calculated as a “virtual” transit time, and a “virtual” travelled distance is derived from body height (12). Alternatively, the heart-to-arm and the heart-to-ankle transit times are determined to calculate the cardio-ankle vascular index (13). Travelled distance from the heart to the ankle is measured on body surface, and heart-to-ankle PWV is calculated. The method is relatively simple, operator-independent, and takes 15-20 minutes. Extreme obesity and arrhythmia may pose problems, as well as peripheral arterial disease.

*Cardio-ankle vascular index*

The cardio-ankle vascular index is less dependent on pressure compared to PWV.

*Aorto-femoral volume wave velocity*

For aorto-femoral volume wave velocity (14), travelled distance is determined from body surface. The measurements are highly reproducible and operator independent.

*Carotid-brachial/radial PWV*

In most studies, tonometry have been used to record the pulse waves.

*Finger to toe pulse wave velocity*

This operator-independent method uses photoplethysmographic probes and the delay between the pulse waves at finger and toe can be calculated as a “virtual” aortic transit time according to the fact that limb arteries are much less elastic than the aorta (15). The travel distance is derived from body height. Results can be obtained within 5 minutes. There is no need to occlude arteries that can modify the global hemodynamics of the vasculature.

*Pulse wave velocity derived with bathroom scales*

An advanced set of bathroom scales can be used to assess PWV at the foot (16) . PWV is estimated from the time delay between ventricular ejection and pulse arrival and an estimate of distance calculated from the subject’s height. The estimated PWV has been found to agree with reference cfPWV (16, 17).

*Brachial pulse pressure*

As compared to invasive BP, systolic BP is often underestimated, and diastolic BP is often overestimated by conventional non-invasive cuff-based BP measurement technique, leading eventually to quite substantial underestimation of true (i.e. invasive) brachial pulse pressure by cuff measurement (18). In any case, validated sphygmomanometers need to be used (19). A brachial pulse pressure >60 mmHg in older people is a sign of increased arterial stiffening, according to the latest version (2018) of the European Society of Cardiology / European Society of Hypertension Guidelines for the management of arterial hypertension (20).

*Central pulse pressure*

Whereas diastolic BP is relatively constant from the aorta to peripheral arteries, systolic BP (and pulse pressure) increases from central to peripheral sites. . Central systolic BP and pulse pressure can be directly read from the resulting waveforms being processed with dedicated formulas (mainly so-called transfer functions) (21). A different method to determine central systolic BP uses a regression, which includes brachial BP, heart rate, body height and the interval between the QRS complex and the last Korotkov sound (22). Determination of central pulse pressure takes 1-2 minutes longer than measurement of brachial pulse pressure, if a fully automated brachial cuff-based system is used, or approximately 10 minutes longer, if waveforms are acquired with tonometry. Cuff-based methods are operator-independent, whereas acquisition of radial (and even more, carotid) pressure waveforms with a tonometer requires some training. However, quality criteria for waveforms are widely established. If cuff-based systems are used, ambulatory 24-hour measurements are feasible.

*Waveform features related to wave reflections*

Based on central waveforms acquired with the methods explained in the previous paragraph, information regarding wave reflection can be obtained from analysis of the curve (pulse waveform analysis) (23). Considering peaks and inflection points, augmentation pressure is the amount of pressure at the level of the ascending aorta, which is attributed to wave reflection, and augmentation index is augmented pressure/central pulse pressure. If (measured or model-based) flow waveforms are available and analysed together with pressure waveforms, wave separation analyses can be performed, yielding amplitudes of the forward and reflected wave and their ratio reflection magnitude. All these analyses are software-based and performed on central waveforms within seconds.

*Photoplethysmographic assessment*

The photoplethysmogram, an optical assessment of blood volume changes, can be used to derive an arterial pulse wave signal at the finger, in a range of devices, including pulse oximeters. Several parameters can be derived from the photoplethysmogram pulse wave, it is non-invasive and an inexpensive method. An association of some of the derived indices with atherosclerosis has been shown (24). Photoplethysmogram is emerging as a potential tool for cardiovascular monitoring in daily life and informing clinical decisions (25).

*Distensibility of large arteries*

Large artery stiffness can be estimated by means of local distensibility. Due to technical reasons, distensibility is most often measured as change in diameter by ultrasound or area by magnetic resonance imaging and peripheral pressure is used. When a reliable local pulse pressure estimation is available, it should be preferred over brachial value, especially when young subjects are studied (8). In both imaging techniques, geometrical changes are generally assessed by semi-automatic tracking of the arterial wall during the cardiac cycle, most often in the carotid artery (ultrasound) or in the aorta (ultrasound and MRI resonance).  Aortic distensibility has been shown to change with age and to predict all-cause mortality and clinical cardiovascular events among individuals without overt cardiovascular disease (26, 27).

*Carotid intima media thickness*

Carotid IMT (28) can be observed at different carotid segments: the common carotid artery far wall is the most commonly used, because it is generally considered better in terms of feasibility, but also carotid bulb and internal carotid are investigated (29) using computerized systems (30). Evaluation is usually obtained on longitudinal scans with linear probe (31, 32). Ultrasound based systems can generally also provide an estimation of carotid elasticity, thus providing both functional and structural parameters of the analysed vessel. B-mode ultrasound is widely used in clinical routine, but (semi-)automated systems for IMT measurement are not.

*Carotid plaque*

Plaques, see definition in main text and (33), often measured via ultrasound, may be characterized by their number, size, irregularity and echodensity (echolucent vs. calcified). Detection of plaques is highly reproducible, operator-dependent but relatively easy, has a good availability, and is currently widely used in clinical routine. Quantitative parameters such as plaque number, thickness, area, and volume can be obtained by non-invasive ultrasound data (34). Plaque phenotypes identification, through the estimation of surface irregularity, echolucency and texture, has been studied (35). When using contrast enhanced ultrasound imaging, based on non-linear response of injected microbubbles to the ultrasound waves, plaque neovascularization, suggestive of plaque instability, can be assessed (36). Besides ultrasound also CT, MRI and nuclear imaging have been used to investigate carotid plaque (37). Detection of carotid plaque received a IIb indication (“may be considered”) in the ESC guidelines of CVD prevention in clinical practice.

*Coronary artery calcium*

Coronary artery calcium (CAC) is a sign of subclinical coronary atherosclerosis (38). A CAC score can be obtained within 15 minutes, but with use of a moderate dose of radiation (mean value 1 mSv) (39). Calculators for arterial age, based on CAC, are available online (40), and already used in clinical practice. The method is highly reproducible, relatively simple and has a moderate availability.

*Ankle-brachial index*

Several methodological recommendations have been made to standardize ankle brachial index (41) measurements. The method is widely available and utilized in clinical routine, albeit mainly for peripheral arterial disease screening. An ankle brachial index < 0.9 is a measure of asymptomatic hypertension-mediated organ damage, according to the recent version of the European Society of Cardiology / European Society of Hypertension Guidelines for the management of arterial hypertension, and also received a IIb indication (“may be considered”) in the ESC Guidelines for CVD prevention in clinical practice.

*Brachial artery flow mediated dilation*

Flow mediated dilation (42, 43) the vasodilatation resulting from the mechanical stimulus. The observed phenomenon is quantified as a percentage increase of the diameter compared to its baseline value. Simultaneous live acquisition of the pulsed-wave Doppler velocity can be used to quantify shear stimulus inducing the vasodilation. Brachial flow mediated dilation is related to the risk of cardiovascular events.

*Large artery inflammation (Positron emission tomography)*

Standardized uptake values represents ^18^F-FDG activity adjusted for ^18^F-fluorodeoxyglucose dose, corrected for decay and divided by body weight. To correct for background ^18^F-fluorodeoxyglucose, whole artery standardized uptake values is either subtracted or divided (target to background ratio) by background standardized uptake values obtained from venous or remote arterial blood.

**3.** **How do vascular ageing measures relate to chronological ageing?**

*Distensibility of large carotid artery*

In children, carotid distensibility increased from <1 to 9 years, and decreased thereafter up to 18 years (44); decreased in boys and to a lesser degree in girls from 6-18 years (45) and decreased from 10-20 years (46); aortic compliance increased from 8-14 years and plateaued thereafter (47). In adults, carotid distensibility coefficient decreased in a non-linear fashion with age in men and women (48) in pooled datasets from several cross-sectional studies.

*Ankle brachial index*

In infants, the ankle brachial index increases from newborns up to 1 year, and plateaus thereafter up to two years (49). In a cross-sectional study (50), ankle brachial index was lowest at <40 years and increased with age by a small amount until 60–69 years and decreased thereafter. In a longitudinal study, the minor increase of ankle brachial index up to 65 years of age and the modest decline thereafter (0.03 per decade) was confirmed, with greater rates at advancing age (51). In both studies, the ankle brachial index was higher in men than in women.

*Aortic diameter*

Ascending aortic diameters increased linearly with age in men and women in cross-sectional analysis (52), starting in children less than 5 years (53).

*Aortic / large artery inflammation*

Fluorine-18 fluorodeoxyglucose (^18^F-FDG) uptake of the aorta and peripheral arteries was measured in a small retrospective study (76 patients undergoing PET/CT due to malignant melanoma) and quantified as tissue-to-background ratio of standardized uptake values. Mean tissue-to-background ratio in the aorta and in peripheral arteries increased exponentially with age (54). In all age groups, the tissue-to-background ratio of the aorta was significantly greater than that of the peripheral arteries.

**6.** **Modification of vascular age by pharmacological intervention**

*Anti-hypertension agents*

Several classes of anti-hypertensive agents are associated with maintaining or improving vascular ageing. These include angiotensin-converting-enzyme inhibitors, angiotensin receptor blockers , calcium channel blockers, some β-blockers (55). Of these, the renin–angiotensin–aldosterone system inhibitors have the greatest effect, as these drugs act on the intrinsic material properties of the arterial wall, rather than through reductions in vascular resistance or cardiac output only. The antioxidant and anti-inflammatory properties promote vasodilation, natriuresis and reduce collagen deposition, which advances endothelial function and improves arterial stiffness (56). Angiotensin-converting-enzyme inhibitors particularly attenuate pulse wave velocity and improve arterial wave reflections (57). Different anti-hypertensive agents exert differential changes in central and peripheral hemodynamics (58), thus selecting treatment decisions on central, rather than brachial pressure is important implications for the modification of vascular ageing.

Recent research has focused on potential novel targets to modify vascular ageing based on the continually increasing mechanistic understanding of the underlying pathophysiology. Examples include anti-inflammatory agents, vascular calcium inhibitors, Nitric oxidedonors, collagen crosslinking inhibitors (or breakdown promoters), mineralocorticoid receptor antagonism, micro-RNA therapy, elastase inhibition, elastin-related peptide-signaling inhibition, micro-RNA therapy and agonists or activators of endogenous calcification inhibitory pathways, such as the matrix glaprotein pathway, as discussed in the 2019 state-of-the-art review by Chirinos et al. (12). Other agents that show favourable vascular results come with unfavorable side effects. An example is the immunosuppressant and anti-proliferative agent rapamycin (mTOR inhibitor), that is reported to have beneficial effects on arterial stiffness, blood pressure, carotid intima media thickness and antiatherosclerotic properties but is also associated with hyperglycemia, hyperlipidemia and insulin resistance (59). Given that the gut microbiome influences inflammation and is inversely associated with cfPWV (60), probiotic use has the potential to modify vascular ageing. A high dose probiotic supplementation for 12 weeks decreased pulse pressure, augmentation index and cfPWV in obese postmenopausal women (61)

*Antihyperlipidaemic agents*

Statin therapy (HMG-CoA reductase inhibitors) produces significant reductions in major vascular events. Agents that target dyslipidaemia, such as statins and PCSK9 inhibitors are effective in atherosclerosis stabilisation and regression (62). Statins have also been effective in reducing arterial stiffness in different populations including those with [hypercholesterolemia](https://www.sciencedirect.com/topics/medicine-and-dentistry/hypercholesterolemia) (63), obesity (64), isolated systolic hypertension (65). The majority of trials show a reduction in cfPWV that is independent of changes in blood pressure (66). Mechanisms to reduce both arterial stiffness and central haemodynamics include anti-inflammatory, anti-oxidative and anti-proliferative pathways beyond their lipid-lowering properties (67). Newer statins have also been shown to reduce the increase in carotid IMT observed over a 10 year period (68).

*Anti-diabetes agents*

Randomised controlled trials assessing the effect of glucose lowering on cardiovascular outcomes have yielded mixed results (69-71). However, new anti-diabetic drugs add cardiovascular protection beyond glycemic control (72-76). These benefits are achieved through both hemodynamic/anti-atherosclerotic mechanisms, and due to improvement of endothelial function, reduced oxidative stress, and improved large artery distensibility (77-80). Example include traditional (Metformin) and newer sodium glucose co-transporter type 2 (SGLT2) inhibitors that improve a variety of vascular ageing measures including, endothelial function, arterial stiffness, forearm-mediated dilation and coronary artery calcification (81). Empagliflozin use on top of metformin treatment can further improve vascular health (82). Insulin treatment mode may also influence arterial stiffness, since lower values were obtained in diabetic patients after continuous subcutaneous insulin infusion, compared to multiple daily injections (83). Chronic insulin therapy was associated with increased cfPWV in hypertensive diabetics (84).

**Supplementary Table 1. Relationship between vascular ageing biomarker with chronological age.**

| **Ageing biomarker** | **Study type** | **Relationship with chronological age** | **Start of change** | **Sex differences** |
| --- | --- | --- | --- | --- |
| Aortic pulse wave velocity | Cross-sectional (53, 85) | Exponential increase | Early childhood | F>M |
| Carotid-femoral pulse wave velocity | Cross-sectional (86-95) | Minor increase in childhood*  Exponential increase adults | Childhood | Minor (M>F) |
|  | Longitudinal (96-98) | Non-linear increase* | 25** years |  |
| Brachial-ankle pulse wave velocity | Cross-sectional and longitudinal (99-101) | Minor increase (M>F)  Exponential increase in adults | 10-14 years | M>F until 58yrs  F>M thereafter |
| Cardio-ankle vascular index | Cross-sectional (102, 103) | Linear increase in children  Exponential increase in adults | 20-29** years | M>F |
| Aorto-femoral volume wave velocity / impedance cardiography | Cross-sectional (14, 104) | Exponential increase | 18** years | M>F |
| Carotid-brachial/ radial pulse wave velocity | Cross-sectional (87, 95, 105, 106) | Minor increase (F) / plateau from 60 years | Childhood | Minor (M>F) |
| Finger-toe pulse wave velocity | Cross-sectional (15) | increase | 20 years |  |
| Estimated aortic pulse wave velocity | Cross-sectional (2, 107, 108) | Non-linear increase in children  Quadratic increase in adults | 8 years | No |
| Pulse wave velocity from bathroom scales | Cross sectional (16) | Increase | Adulthood | NA |
| Brachial pulse pressure | Cross-sectional (107, 109, 110) | Increase (M) / marginal decrease (F) in childhood  Small decrease (M) / plateau (F) young adults  Exponential increase after middle age (F>M) | Childhood (M)  40-50 years | M>F until 40-50 yrs  F> M thereafter |
|  | Longitudinal (96, 109) | Small decrease (M) / plateau (F) young adults  Exponential increase after 40 years (F>M)  Decrease in elderly M | 40** years | M>F until 40yrs  F>M thereafter |
| Central pulse pressure | Cross-sectional (107, 111) | Linear increase in children (M>F)  Exponential increase in adults | Childhood | M>F in young age  F=M thereafter |
| Augmentation index | Cross-sectional (87, 105, 107, 111-115) | Decrease in children up to 15  Logistic increase in adults | Childhood | W>F |
| Augmentation pressure | Cross-sectional (87, 107) | Linear increase  Plateau elderly M | 18** years | F>M |
| Backward wave amplitude | Cross-sectional (106, 107) | Exponential increase  Decrease elderly F | 18** years | Minor |
|  | Longitudinal (97) | Decrease | 35** years | Minor |
| Reflection magnitude | Cross-sectional (107) | Inverse exponential increase | 18 years | F>M |
|  | Longitudinal (97) | Decrease | 35** years | Minor |
| Photoplethysmogram-based reflection index | Cross-sectional (116) | Linear increase | 30 years | NA |
| Carotid distensibility | Cross-sectional (44-48) | Increase from < 1 to 4 years, small increase or decrease in children thereafter  Decrease in adults | 6 years | F>M  (in adults) |
| Carotid intima media thickness | Cross-sectional (44-47, 111, 117-119) | Minor non-linear or linear increase in children  Linear increase in adults | <1 year | M>F (minor) |
| Carotid plaque | Cross-sectional (119, 120) | Exponential increase | 40-50* years (M)  menopause (F) | M > F before menopause, minor differences thereafter |
|  | Longitudinal (120) | Inverse exponential increase | 40-50* years (M)  menopause (F) |  |
| Coronary artery calcium | Cross-sectional (121, 122) | Exponential increase | 45** years | M>F |
|  | Longitudinal (123) | Exponential increase | 45** years | M>F |
| Ankle brachial index | Cross-sectional (49, 50) | Increase from newborns to year 2; Minor increase – 69 years then minor decrease | newborns | M>F |
|  | Longitudinal (51) | Minor increase – 65 years, plateau – 70 years, decrease thereafter | 50 years | M>F |
| Brachial artery flow mediated dilation | Cross-sectional (44, 124-128) | Small increase from 8-14 years or decrease from 10-18 years, thereafter decrease in children and adults | 8 years | F>M |
| Aortic diameter | Cross-sectional (52, 53) | Non-linear (children)  Linear (adults) | 1 year | M=F (childhood)  M>F (adults) |
| Aortic/large artery inflammation (PET) | Cross-sectional (129) | Exponential | 20 years* | NA |

M, male; F, female; NA, not available.

*the relationship between males and females is not consistent

**no data in early life.

**Supplementary References**

1. Weber T, Maas R, Auer J, Lamm G, Lassnig E, Rammer M, et al. Arterial wave reflections and determinants of endothelial function a hypothesis based on peripheral mode of action. Am J Hypertens. 2007;20(3):256-62.

2. Weber T, Wassertheurer S, Hametner B, Parragh S, Eber B. Noninvasive methods to assess pulse wave velocity: comparison with the invasive gold standard and relationship with organ damage. J Hypertens. 2015;33(5):1023-31.

3. Hametner B, Wassertheurer S, Mayer CC, Danninger K, Binder RK, Weber T. Aortic Pulse Wave Velocity Predicts Cardiovascular Events and Mortality in Patients Undergoing Coronary Angiography: A Comparison of Invasive Measurements and Noninvasive Estimates. Hypertension. 2021;77(2):571-81.

4. Wentland AL, Wieben O, François CJ, Boncyk C, Munoz Del Rio A, Johnson KM, et al. Aortic pulse wave velocity measurements with undersampled 4D flow‐sensitive MRI: comparison with 2D and algorithm determination. Journal of Magnetic Resonance Imaging. 2013;37(4):853-9.

5. Guala A, Rodriguez-Palomares J, Dux-Santoy L, Teixido-Tura G, Maldonado G, Galian L, et al. Influence of aortic dilation on the regional aortic stiffness of bicuspid aortic valve assessed by 4-dimensional flow cardiac magnetic resonance: comparison with Marfan syndrome and degenerative aortic aneurysm. JACC: Cardiovascular Imaging. 2019;12(6):1020-9.

6. Van Engelen A, Vieira MS, Rafiq I, Cecelja M, Schneider T, De Bliek H, et al. Aortic length measurements for pulse wave velocity calculation: manual 2D vs automated 3D centreline extraction. Journal of Cardiovascular Magnetic Resonance. 2017;19(1):1-13.

7. Bargiotas I, Mousseaux E, Yu W-C, Venkatesh BA, Bollache E, De Cesare A, et al. Estimation of aortic pulse wave transit time in cardiovascular magnetic resonance using complex wavelet cross-spectrum analysis. Journal of Cardiovascular Magnetic Resonance. 2015;17(1):1-11.

8. Laurent S, Cockcroft J, Van Bortel L, Boutouyrie P, Giannattasio C, Hayoz D, et al. Expert consensus document on arterial stiffness: methodological issues and clinical applications. European heart journal. 2006;27(21):2588-605.

9. Vlachopoulos C, Xaplanteris P, Aboyans V, Brodmann M, Cífková R, Cosentino F, et al. The role of vascular biomarkers for primary and secondary prevention. A position paper from the European Society of Cardiology Working Group on peripheral circulation: Endorsed by the Association for Research into Arterial Structure and Physiology (ARTERY) Society. Atherosclerosis. 2015;241(2):507-32.

10. Townsend RR, Wilkinson IB, Schiffrin EL, Avolio AP, Chirinos JA, Cockcroft JR, et al. Recommendations for improving and standardizing vascular research on arterial stiffness: a scientific statement from the American Heart Association. Hypertension. 2015;66(3):698-722.

11. Weir-McCall JR, Brown L, Summersgill J, Talarczyk P, Bonnici-Mallia M, Chin SC, et al. Development and validation of a path length calculation for carotid–femoral pulse wave velocity measurement: a TASCFORCE, SUMMIT, and Caerphilly collaborative venture. Hypertension. 2018;71(5):937-45.

12. Chirinos JA, Segers P, Hughes T, Townsend R. Large-Artery Stiffness in Health and Disease: JACC State-of-the-Art Review. Journal of the American College of Cardiology. 2019;74(9):1237-63.

13. Shirai K, Hiruta N, Song M, Kurosu T, Suzuki J, Tomaru T, et al. Cardio-ankle vascular index (CAVI) as a novel indicator of arterial stiffness: theory, evidence and perspectives. Journal of atherosclerosis and thrombosis. 2011:1105300383-.

14. Skrabal F, Weber T, Skrabal K, Windhaber J, Ehsas H, Stockinger N, et al. Measurement of aortofemoral volume wave velocity during the routine 12-channel ECG: relation to age, physiological hemoglobin A 1C, triglycerides and SBP in healthy individuals. Journal of hypertension. 2020;38(10):1989-99.

15. Hallab M, Pichierri S, Boin J, Tramblay M, Chevalet P, Berrut G, editors. A new index to evaluate arterial ageing independently of arterial blood pressure: pOpscore (®). Annales de Cardiologie et D'angeiologie; 2012.

16. Campo D, Khettab H, Yu R, Genain N, Edouard P, Buard N, et al. Measurement of aortic pulse wave velocity with a connected bathroom scale. American journal of hypertension. 2017;30(9):876-83.

17. Collier SR, McCraw C, Campany M, Lubkeman A, StClair P, Ji H, et al. Withings Body Cardio versus Gold Standards of Pulse-Wave Velocity and Body Composition. Journal of personalized medicine. 2020;10(1):17.

18. Picone DS, Schultz MG, Otahal P, Aakhus S, Al-Jumaily AM, Black JA, et al. Accuracy of cuff-measured blood pressure: systematic reviews and meta-analyses. Journal of the American College of Cardiology. 2017;70(5):572-86.

19. Stergiou GS, Asmar R, Myers M, Palatini P, Parati G, Shennan A, et al. Improving the accuracy of blood pressure measurement: the influence of the European Society of Hypertension International Protocol (ESH-IP) for the validation of blood pressure measuring devices and future perspectives. LWW; 2018.

20. Williams B, Mancia G, Spiering W, Agabiti Rosei E, Azizi M, Burnier M, et al. 2018 ESC/ESH Guidelines for the management of arterial hypertension: The Task Force for the management of arterial hypertension of the European Society of Cardiology (ESC) and the European Society of Hypertension (ESH). European heart journal. 2018;39(33):3021-104.

21. Gallagher D, Adji A, O’Rourke MF. Validation of the transfer function technique for generating central from peripheral upper limb pressure waveform. American Journal of Hypertension. 2004;17(11):1059-67.

22. Cremer A, Butlin M, Codjo L, Coulon P, Ranouil X, Joret C, et al. Determination of central blood pressure by a noninvasive method (brachial BP and QKD interval). Journal of hypertension. 2012;30(8):1533-9.

23. O'Rourke MF, Gallagher DE. Pulse wave analysis. Journal of hypertension Supplement: official journal of the International Society of Hypertension. 1996;14(5):S147-57.

24. Peltokangas M, Vehkaoja A, Huotari M, Verho J, Mattila VM, Röning J, et al. Combining finger and toe photoplethysmograms for the detection of atherosclerosis. Physiological measurement. 2017;38(2):139.

25. Charlton PH, Kyriacou PA, Mant J, Marozas V, Chowienczyk P, Alastruey J. Wearable photoplethysmography for cardiovascular monitoring. Proceedings of the IEEE. 2022;110(3):355-81.

26. Redheuil A, Wu CO, Kachenoura N, Ohyama Y, Yan RT, Bertoni AG, et al. Proximal aortic distensibility is an independent predictor of all-cause mortality and incident CV events: the MESA study. Journal of the American College of Cardiology. 2014;64(24):2619-29.

27. Maroules CD, Khera A, Ayers C, Goel A, Peshock RM, Abbara S, et al. Cardiovascular outcome associations among cardiovascular magnetic resonance measures of arterial stiffness: the Dallas heart study. Journal of Cardiovascular Magnetic Resonance. 2014;16(1):1-9.

28. Touboul P-J, Hennerici M, Meairs S, Adams H, Amarenco P, Bornstein N, et al. Mannheim carotid intima-media thickness consensus (2004–2006). Cerebrovascular diseases. 2007;23(1):75-80.

29. Bianchini E, Giannarelli C, Maria Bruno R, Armenia S, Landini L, Faita F, et al. Functional and structural alterations of large arteries: methodological issues. Current pharmaceutical design. 2013;19(13):2390-400.

30. Molinari F, Zeng G, Suri JS. A state of the art review on intima–media thickness (IMT) measurement and wall segmentation techniques for carotid ultrasound. Computer methods and programs in biomedicine. 2010;100(3):201-21.

31. Bianchini E, Bozec E, Gemignani V, Faita F, Giannarelli C, Ghiadoni L, et al. Assessment of carotid stiffness and intima‐media thickness from ultrasound data: comparison between two methods. Journal of Ultrasound in Medicine. 2010;29(8):1169-75.

32. Potter K, Reed CJ, Green DJ, Hankey GJ, Arnolda LF. Ultrasound settings significantly alter arterial lumen and wall thickness measurements. Cardiovascular ultrasound. 2008;6(1):1-11.

33. Members ATF, Piepoli MF, Hoes AW, Agewall S, Albus C, Brotons C, et al. 2016 European Guidelines on cardiovascular disease prevention in clinical practice: The Sixth Joint Task Force of the European Society of Cardiology and Other Societies on Cardiovascular Disease Prevention in Clinical Practice (constituted by representatives of 10 societies and by invited experts) Developed with the special contribution of the European Association for Cardiovascular Prevention & Rehabilitation (EACPR). European journal of preventive cardiology. 2016;23(11):NP1-NP96.

34. Naqvi TZ, Lee M-S. Carotid intima-media thickness and plaque in cardiovascular risk assessment. JACC: Cardiovascular Imaging. 2014;7(10):1025-38.

35. Prati P, Tosetto A, Casaroli M, Bignamini A, Canciani L, Bornstein N, et al. Carotid plaque morphology improves stroke risk prediction: usefulness of a new ultrasonographic score. Cerebrovascular diseases. 2011;31(3):300-4.

36. Schinkel AF, Bosch JG, Staub D, Adam D, Feinstein SB. Contrast-enhanced ultrasound to assess carotid intraplaque neovascularization. Ultrasound in medicine & biology. 2020;46(3):466-78.

37. Daghem M, Bing R, Fayad ZA, Dweck MR. Noninvasive imaging to assess atherosclerotic plaque composition and disease activity: coronary and carotid applications. Cardiovascular Imaging. 2020;13(4):1055-68.

38. McClelland RL, Jorgensen NW, Budoff M, Blaha MJ, Post WS, Kronmal RA, et al. 10-year coronary heart disease risk prediction using coronary artery calcium and traditional risk factors: derivation in the MESA (Multi-Ethnic Study of Atherosclerosis) with validation in the HNR (Heinz Nixdorf Recall) study and the DHS (Dallas Heart Study). Journal of the American College of Cardiology. 2015;66(15):1643-53.

39. Patel AA, Fine J, Naghavi M, Budoff MJ. Radiation exposure and coronary artery calcium scans in the society for heart attack prevention and eradication cohort. The international journal of cardiovascular imaging. 2019;35(1):179-83.

40. McClelland RL, Nasir K, Budoff M, Blumenthal RS, Kronmal RA. Arterial age as a function of coronary artery calcium (from the Multi-Ethnic Study of Atherosclerosis [MESA]). The American journal of cardiology. 2009;103(1):59-63.

41. Aboyans V, Criqui MH, Abraham P, Allison MA, Creager MA, Diehm C, et al. Measurement and interpretation of the ankle-brachial index: a scientific statement from the American Heart Association. Circulation. 2012;126(24):2890-909.

42. Celermajer DS, Sorensen KE, Gooch V, Spiegelhalter D, Miller O, Sullivan I, et al. Non-invasive detection of endothelial dysfunction in children and adults at risk of atherosclerosis. The lancet. 1992;340(8828):1111-5.

43. Corretti MC, Anderson TJ, Benjamin EJ, Celermajer D, Charbonneau F, Creager MA, et al. Guidelines for the ultrasound assessment of endothelial-dependent flow-mediated vasodilation of the brachial artery: a report of the International Brachial Artery Reactivity Task Force. Journal of the American College of Cardiology. 2002;39(2):257-65.

44. Sarkola T, Manlhiot C, Slorach C, Bradley TJ, Hui W, Mertens L, et al. Evolution of the arterial structure and function from infancy to adolescence is related to anthropometric and blood pressure changes. Arteriosclerosis, thrombosis, and vascular biology. 2012;32(10):2516-24.

45. Doyon A, Kracht D, Bayazit AK, Deveci M, Duzova A, Krmar RT, et al. Carotid artery intima-media thickness and distensibility in children and adolescents: reference values and role of body dimensions. Hypertension. 2013;62(3):550-6.

46. Jourdan C, Wühl E, Litwin M, Fahr K, Trelewicz J, Jobs K, et al. Normative values for intima–media thickness and distensibility of large arteries in healthy adolescents. Journal of hypertension. 2005;23(9):1707-15.

47. Weberruß H, Pirzer R, Böhm B, Elmenhorst J, Dalla Pozza R, Netz H, et al. Increased intima-media thickness is not associated with stiffer arteries in children. Atherosclerosis. 2015;242(1):48-55.

48. Engelen L, Bossuyt J, Ferreira I, van Bortel LM, Reesink KD, Segers P, et al. Reference values for local arterial stiffness. Part A: carotid artery. Journal of hypertension. 2015;33(10):1981-96.

49. Katz S, Globerman A, Avitzour M, Dolfin T. The ankle-brachial index in normal neonates and infants is significantly lower than in older children and adults. Journal of pediatric surgery. 1997;32(2):269-71.

50. Ishida A, Miyagi M, Kinjo K, Ohya Y. Age- and sex-related effects on ankle-brachial index in a screened cohort of Japanese: the Okinawa Peripheral Arterial Disease Study (OPADS). European journal of preventive cardiology. 2014;21(6):712-8.

51. Oberdier MT, Morrell CH, Lakatta EG, Ferrucci L, AlGhatrif M. Subclinical Longitudinal Change in Ankle-Brachial Index With Aging in a Community-Dwelling Population Is Associated With Central Arterial Stiffening. Journal of the American Heart Association. 2019;8(15):e011650.

52. Turkbey EB, Jain A, Johnson C, Redheuil A, Arai AE, Gomes AS, et al. Determinants and normal values of ascending aortic diameter by age, gender, and race/ethnicity in the Multi-Ethnic Study of Atherosclerosis (MESA). Journal of magnetic resonance imaging : JMRI. 2014;39(2):360-8.

53. Voges I, Jerosch-Herold M, Hedderich J, Pardun E, Hart C, Gabbert DD, et al. Normal values of aortic dimensions, distensibility, and pulse wave velocity in children and young adults: a cross-sectional study. Journal of Cardiovascular Magnetic Resonance. 2012;14(1):1-13.

54. Pasha AK, Moghbel M, Saboury B, Gharavi MH, Blomberg BA, Torigian DA, et al. Effects of age and cardiovascular risk factors on (18) F-FDG PET/CT quantification of atherosclerosis in the aorta and peripheral arteries. Hellenic journal of nuclear medicine. 2015;18(1):5-10.

55. Janić M, Lunder M, Sabovič M. Arterial stiffness and cardiovascular therapy. Biomed Res Int. 2014;2014:621437.

56. Neves MF, Cunha AR, Cunha MR, Gismondi RA, Oigman W. The Role of Renin-Angiotensin-Aldosterone System and Its New Components in Arterial Stiffness and Vascular Aging. High Blood Press Cardiovasc Prev. 2018;25(2):137-45.

57. Shahin Y, Khan JA, Chetter I. Angiotensin converting enzyme inhibitors effect on arterial stiffness and wave reflections: a meta-analysis and meta-regression of randomised controlled trials. Atherosclerosis. 2012;221(1):18-33.

58. McEniery CM, Cockcroft JR, Roman MJ, Franklin SS, Wilkinson IB. Central blood pressure: current evidence and clinical importance. European heart journal. 2014;35(26):1719-25.

59. Salmon AB. About-face on the metabolic side effects of rapamycin. Oncotarget. 2015;6(5):2585-6.

60. Menni C, Lin C, Cecelja M, Mangino M, Matey-Hernandez ML, Keehn L, et al. Gut microbial diversity is associated with lower arterial stiffness in women. European heart journal. 2018;39(25):2390-7.

61. Szulińska M, Łoniewski I, Skrypnik K, Sobieska M, Korybalska K, Suliburska J, et al. Multispecies Probiotic Supplementation Favorably Affects Vascular Function and Reduces Arterial Stiffness in Obese Postmenopausal Women-A 12-Week Placebo-Controlled and Randomized Clinical Study. Nutrients. 2018;10(11).

62. Libby P. The changing landscape of atherosclerosis. Nature. 2021;592(7855):524-33.

63. Pirro M, Schillaci G, Mannarino MR, Savarese G, Vaudo G, Siepi D, et al. Effects of rosuvastatin on 3-nitrotyrosine and aortic stiffness in hypercholesterolemia. Nutr Metab Cardiovasc Dis. 2007;17(6):436-41.

64. Orr JS, Dengo AL, Rivero JM, Davy KP. Arterial destiffening with atorvastatin in overweight and obese middle-aged and older adults. Hypertension. 2009;54(4):763-8.

65. Ferrier KE, Muhlmann MH, Baguet JP, Cameron JD, Jennings GL, Dart AM, et al. Intensive cholesterol reduction lowers blood pressure and large artery stiffness in isolated systolic hypertension. Journal of the American College of Cardiology. 2002;39(6):1020-5.

66. Nowak KL, Rossman MJ, Chonchol M, Seals DR. Strategies for Achieving Healthy Vascular Aging. Hypertension. 2018;71(3):389-402.

67. Kanaki AI, Sarafidis PA, Georgianos PI, Kanavos K, Tziolas IM, Zebekakis PE, et al. Effects of low-dose atorvastatin on arterial stiffness and central aortic pressure augmentation in patients with hypertension and hypercholesterolemia. American journal of hypertension. 2013;26(5):608-16.

68. Lind L. Effect of new statin treatment on carotid artery intima-media thickness: A real-life observational study over 10 years. Atherosclerosis. 2020;306:6-10.

69. Patel A, MacMahon S, Chalmers J, Neal B, Billot L, Woodward M, et al. Intensive blood glucose control and vascular outcomes in patients with type 2 diabetes. N Engl J Med. 2008;358(24):2560-72.

70. Gerstein HC, Miller ME, Byington RP, Goff DC, Jr., Bigger JT, Buse JB, et al. Effects of intensive glucose lowering in type 2 diabetes. N Engl J Med. 2008;358(24):2545-59.

71. Scirica BM, Bhatt DL, Braunwald E, Steg PG, Davidson J, Hirshberg B, et al. Saxagliptin and cardiovascular outcomes in patients with type 2 diabetes mellitus. N Engl J Med. 2013;369(14):1317-26.

72. Laurent S, Boutouyrie P, Cunha PG, Lacolley P, Nilsson PM. Concept of Extremes in Vascular Aging. Hypertension. 2019;74(2):218-28.

73. Zinman B, Wanner C, Lachin JM, Fitchett D, Bluhmki E, Hantel S, et al. Empagliflozin, Cardiovascular Outcomes, and Mortality in Type 2 Diabetes. The New England journal of medicine. 2015;373(22):2117-28.

74. Marso SP, Daniels GH, Brown-Frandsen K, Kristensen P, Mann JF, Nauck MA, et al. Liraglutide and Cardiovascular Outcomes in Type 2 Diabetes. N Engl J Med. 2016;375(4):311-22.

75. Pfeffer MA, Claggett B, Diaz R, Dickstein K, Gerstein HC, Køber LV, et al. Lixisenatide in Patients with Type 2 Diabetes and Acute Coronary Syndrome. N Engl J Med. 2015;373(23):2247-57.

76. Paneni F, Lüscher TF. Cardiovascular Protection in the Treatment of Type 2 Diabetes: A Review of Clinical Trial Results Across Drug Classes. Am J Cardiol. 2017;120(1s):S17-s27.

77. Cherney DZ, Perkins BA, Soleymanlou N, Har R, Fagan N, Johansen OE, et al. The effect of empagliflozin on arterial stiffness and heart rate variability in subjects with uncomplicated type 1 diabetes mellitus. Cardiovasc Diabetol. 2014;13:28.

78. Lambadiari V, Pavlidis G, Kousathana F, Varoudi M, Vlastos D, Maratou E, et al. Effects of 6-month treatment with the glucagon like peptide-1 analogue liraglutide on arterial stiffness, left ventricular myocardial deformation and oxidative stress in subjects with newly diagnosed type 2 diabetes. Cardiovasc Diabetol. 2018;17(1):8.

79. Solini A, Giannini L, Seghieri M, Vitolo E, Taddei S, Ghiadoni L, et al. Dapagliflozin acutely improves endothelial dysfunction, reduces aortic stiffness and renal resistive index in type 2 diabetic patients: a pilot study. Cardiovasc Diabetol. 2017;16(1):138.

80. Batzias K, Antonopoulos AS, Oikonomou E, Siasos G, Bletsa E, Stampouloglou PK, et al. Effects of Newer Antidiabetic Drugs on Endothelial Function and Arterial Stiffness: A Systematic Review and Meta-Analysis. J Diabetes Res. 2018;2018:1232583.

81. Striepe K, Jumar A, Ott C, Karg MV, Schneider MP, Kannenkeril D, et al. Effects of the Selective Sodium-Glucose Cotransporter 2 Inhibitor Empagliflozin on Vascular Function and Central Hemodynamics in Patients With Type 2 Diabetes Mellitus. Circulation. 2017;136(12):1167-9.

82. Lunder M, Janić M, Japelj M, Juretič A, Janež A, Šabovič M. Empagliflozin on top of metformin treatment improves arterial function in patients with type 1 diabetes mellitus. Cardiovasc Diabetol. 2018;17(1):153.

83. Rosenlund S, Theilade S, Hansen TW, Andersen S, Rossing P. Treatment with continuous subcutaneous insulin infusion is associated with lower arterial stiffness. Acta Diabetol. 2014;51(6):955-62.

84. Agnoletti D, Lieber A, Zhang Y, Protogerou AD, Borghi C, Blacher J, et al. Central hemodynamic modifications in diabetes mellitus. Atherosclerosis. 2013;230(2):315-21.

85. Redheuil A, Yu WC, Wu CO, Mousseaux E, de Cesare A, Yan R, et al. Reduced ascending aortic strain and distensibility: earliest manifestations of vascular aging in humans. Hypertension. 2010;55(2):319-26.

86. Reference Values for Arterial Stiffness C. Determinants of pulse wave velocity in healthy people and in the presence of cardiovascular risk factors: 'establishing normal and reference values'. European heart journal. 2010;31(19):2338-50.

87. McEniery CM, Yasmin, Hall IR, Qasem A, Wilkinson IB, Cockcroft JR, et al. Normal vascular aging: differential effects on wave reflection and aortic pulse wave velocity: the Anglo-Cardiff Collaborative Trial (ACCT). Journal of the American College of Cardiology. 2005;46(9):1753-60.

88. Avolio AP, Chen SG, Wang RP, Zhang CL, Li MF, O'Rourke MF. Effects of aging on changing arterial compliance and left ventricular load in a northern Chinese urban community. Circulation. 1983;68(1):50-8.

89. Avolio AP, Deng FQ, Li WQ, Luo YF, Huang ZD, Xing LF, et al. Effects of aging on arterial distensibility in populations with high and low prevalence of hypertension: comparison between urban and rural communities in China. Circulation. 1985;71(2):202-10.

90. Fischer DC, Schreiver C, Heimhalt M, Noerenberg A, Haffner D. Pediatric reference values of carotid-femoral pulse wave velocity determined with an oscillometric device. Journal of hypertension. 2012;30(11):2159-67.

91. Thurn D, Doyon A, Sozeri B, Bayazit AK, Canpolat N, Duzova A, et al. Aortic Pulse Wave Velocity in Healthy Children and Adolescents: Reference Values for the Vicorder Device and Modifying Factors. American journal of hypertension. 2015;28(12):1480-8.

92. Silva AB, Capingana DP, Magalhaes P, Molina Mdel C, Baldo MP, Mill JG. Predictors and Reference Values of Pulse Wave Velocity in Prepubertal Angolan Children. J Clin Hypertens (Greenwich). 2016;18(8):725-32.

93. Mora-Urda AI, Molina MD, Mill JG, Montero-Lopez P. Carotid-Femoral Pulse Wave Velocity in Healthy Spanish Children: Reference Percentile Curves. J Clin Hypertens (Greenwich). 2017;19(3):227-34.

94. Reusz GS, Cseprekal O, Temmar M, Kis E, Cherif AB, Thaleb A, et al. Reference values of pulse wave velocity in healthy children and teenagers. Hypertension. 2010;56(2):217-24.

95. Bia D, Zócalo Y. Physiological age-and sex-related profiles for local (aortic) and regional (carotid-femoral, carotid-radial) pulse wave velocity and center-to-periphery stiffness gradient, with and without blood pressure adjustments: reference intervals and agreement between methods in healthy subjects (3–84 years). Journal of cardiovascular development and disease. 2021;8(1):3.

96. Scuteri A, Morrell CH, Orru M, Strait JB, Tarasov KV, Ferreli LA, et al. Longitudinal perspective on the conundrum of central arterial stiffness, blood pressure, and aging. Hypertension. 2014;64(6):1219-27.

97. Campos-Arias D, De Buyzere ML, Chirinos JA, Rietzschel ER, Segers P. Longitudinal Changes of Input Impedance, Pulse Wave Velocity, and Wave Reflection in a Middle-Aged Population: The Asklepios Study. Hypertension. 2021:HYPERTENSIONAHA12016149.

98. AlGhatrif M, Strait JB, Morrell CH, Canepa M, Wright J, Elango P, et al. Longitudinal trajectories of arterial stiffness and the role of blood pressure: the Baltimore Longitudinal Study of Aging. Hypertension. 2013;62(5):934-41.

99. Lu Y, Pechlaner R, Cai J, Yuan H, Huang Z, Yang G, et al. Trajectories of Age-Related Arterial Stiffness in Chinese Men and Women. Journal of the American College of Cardiology. 2020;75(8):870-80.

100. Niboshi A, Hamaoka K, Sakata K, Inoue F. Characteristics of brachial-ankle pulse wave velocity in Japanese children. Eur J Pediatr. 2006;165(9):625-9.

101. Collins RT, Somes GW, Alpert BS. Differences in arterial compliance among normotensive adolescent groups: Collins arterial compliance in adolescents. Pediatr Cardiol. 2008;29(5):929-34.

102. Shirai K, Suzuki K, Tsuda S, Shimizu K, Takata M, Yamamoto T, et al. Comparison of Cardio-Ankle Vascular Index (CAVI) and CAVI0 in Large Healthy and Hypertensive Populations. Journal of atherosclerosis and thrombosis. 2019;26(7):603-15.

103. Jurko T, Mestanik M, Jurko A, Jr., Spronck B, Avolio A, Mestanikova A, et al. Pediatric reference values for arterial stiffness parameters cardio-ankle vascular index and CAVI0. Journal of the American Society of Hypertension : JASH. 2018;12(11):e35-e43.

104. Koivistoinen T, Koobi T, Jula A, Hutri-Kahonen N, Raitakari OT, Majahalme S, et al. Pulse wave velocity reference values in healthy adults aged 26-75 years. Clin Physiol Funct Imaging. 2007;27(3):191-6.

105. Mitchell GF, Wang N, Palmisano JN, Larson MG, Hamburg NM, Vita JA, et al. Hemodynamic correlates of blood pressure across the adult age spectrum: noninvasive evaluation in the Framingham Heart Study. Circulation. 2010;122(14):1379-86.

106. Mitchell GF, Parise H, Benjamin EJ, Larson MG, Keyes MJ, Vita JA, et al. Changes in arterial stiffness and wave reflection with advancing age in healthy men and women: the Framingham Heart Study. Hypertension. 2004;43(6):1239-45.

107. Weber T, Wassertheurer S, Hametner B, Moebus S, Pundt N, Mahabadi AA, et al. Cross-sectional analysis of pulsatile hemodynamics across the adult life span: reference values, healthy and early vascular aging: the Heinz Nixdorf Recall and the MultiGeneration Study. Journal of hypertension. 2019;37(12):2404-13.

108. Elmenhorst J, Hulpke-Wette M, Barta C, Dalla Pozza R, Springer S, Oberhoffer R. Percentiles for central blood pressure and pulse wave velocity in children and adolescents recorded with an oscillometric device. Atherosclerosis. 2015;238(1):9-16.

109. Ji H, Kim A, Ebinger JE, Niiranen TJ, Claggett BL, Bairey Merz CN, et al. Sex Differences in Blood Pressure Trajectories Over the Life Course. JAMA Cardiol. 2020;5(3):19-26.

110. Zachariah JP, Graham DA, de Ferranti SD, Vasan RS, Newburger JW, Mitchell GF. Temporal trends in pulse pressure and mean arterial pressure during the rise of pediatric obesity in US children. Journal of the American Heart Association. 2014;3(3):e000725.

111. Curcio S, García-Espinosa V, Arana M, Farro I, Chiesa P, Giachetto G, et al. Growing-related changes in arterial properties of healthy children, adolescents, and young adults nonexposed to cardiovascular risk factors: analysis of gender-related differences. International journal of hypertension. 2016;2016.

112. Hidvegi EV, Illyes M, Molnar FT, Cziraki A. Influence of body height on aortic systolic pressure augmentation and wave reflection in childhood. Journal of human hypertension. 2015;29(8):495-501.

113. Zaniqueli D, Baldo MP, Sartorio CL, de Sa Cunha R, de Oliveira Alvim R, Mill JG. Early sex differences in central arterial wave reflection are mediated by different timing of forward and reflected pressure waves. Clinical and experimental pharmacology & physiology. 2018;45(2):166-73.

114. Diaz A, Zócalo Y, Bia D, Cabrera Fischer E. Reference Intervals of central aortic blood pressure and augmentation index assessed with an oscillometric device in healthy children, adolescents, and young adults from Argentina. International journal of hypertension. 2018;2018.

115. Murakami T, Takeda A, Takei K, Ueno M, Yakuwa S, Yamazawa H, et al. Aortic pressure wave reflection in children. Hypertension Research. 2010;33(3):225-8.

116. Yousef Q RM, Ali MAM. The Analysis of PPG Morphology: Investigating the Effects of Aging on Arterial Compliance. MEASUREMENT SCIENCE REVIEW,. 2012;12(6):266-71.

117. Engelen L, Ferreira I, Stehouwer CD, Boutouyrie P, Laurent S, Reference Values for Arterial Measurements C. Reference intervals for common carotid intima-media thickness measured with echotracking: relation with risk factors. European heart journal. 2013;34(30):2368-80.

118. Scuteri A, Najjar SS, Muller DC, Andres R, Hougaku H, Metter EJ, et al. Metabolic syndrome amplifies the age-associated increases in vascular thickness and stiffness. Journal of the American College of Cardiology. 2004;43(8):1388-95.

119. Homma S, Hirose N, Ishida H, Ishii T, Araki G. Carotid plaque and intima-media thickness assessed by b-mode ultrasonography in subjects ranging from young adults to centenarians. Stroke; a journal of cerebral circulation. 2001;32(4):830-5.

120. Kiechl S, Willeit J. The natural course of atherosclerosis. Part I: incidence and progression. Arteriosclerosis, thrombosis, and vascular biology. 1999;19(6):1484-90.

121. McClelland RL, Chung H, Detrano R, Post W, Kronmal RA. Distribution of coronary artery calcium by race, gender, and age: results from the Multi-Ethnic Study of Atherosclerosis (MESA). Circulation. 2006;113(1):30-7.

122. Schmermund A, Mohlenkamp S, Berenbein S, Pump H, Moebus S, Roggenbuck U, et al. Population-based assessment of subclinical coronary atherosclerosis using electron-beam computed tomography. Atherosclerosis. 2006;185(1):177-82.

123. Erbel R, Lehmann N, Churzidse S, Rauwolf M, Mahabadi AA, Mohlenkamp S, et al. Progression of coronary artery calcification seems to be inevitable, but predictable - results of the Heinz Nixdorf Recall (HNR) study. European heart journal. 2014;35(42):2960-71.

124. Celermajer DS, Sorensen KE, Spiegelhalter DJ, Georgakopoulos D, Robinson J, Deanfield JE. Aging is associated with endothelial dysfunction in healthy men years before the age-related decline in women. Journal of the American College of Cardiology. 1994;24(2):471-6.

125. Skaug EA, Aspenes ST, Oldervoll L, Morkedal B, Vatten L, Wisloff U, et al. Age and gender differences of endothelial function in 4739 healthy adults: the HUNT3 Fitness Study. European journal of preventive cardiology. 2013;20(4):531-40.

126. Juonala M, Kahonen M, Laitinen T, Hutri-Kahonen N, Jokinen E, Taittonen L, et al. Effect of age and sex on carotid intima-media thickness, elasticity and brachial endothelial function in healthy adults: the cardiovascular risk in Young Finns Study. European heart journal. 2008;29(9):1198-206.

127. Konigstein K, Wagner J, Frei M, Knaier R, Klenk C, Carrard J, et al. Endothelial function of healthy adults from 20 to 91 years of age: prediction of cardiovascular risk by vasoactive range. Journal of hypertension. 2021.

128. Li A, Celermajer D, Chan M, Sung R, Woo K. Reference range for brachial artery flow-mediated dilation in healthy Chinese children and adolescents. Hong Kong Med J. 2018;24(Suppl 3):36-8.

129. Pasha AK, Moghbel M, Saboury B, Gharavi MH, Blomberg BA, Torigian DA, et al. Effects of age and cardiovascular risk factors on (18)F-FDG PET/CT quantification of atherosclerosis in the aorta and peripheral arteries. Hell J Nucl Med. 2015;18(1):5-10.
